# Linnett is Back: Chemical Bonding through the Lens of Born Maxima

**DOI:** 10.1021/acs.jctc.4c01785

**Published:** 2025-02-21

**Authors:** María Menéndez-Herrero, Evelio Francisco, Ángel Martín Pendás

**Affiliations:** Departamento de Química Física y Analítica. Facultad de Química, 201467 Universidad de Oviedo, 33006 Oviedo, Spain

## Abstract

The classical Lewis–Langmuir electron pair model remains central to chemical bonding theories despite its inherent contradictions with quantum mechanical principles such as antisymmetry. This paper revisits the long-forgotten Linnett’s double quartet (LDQ) model, which integrates spin considerations into chemical bonding. We demonstrate that the distribution of electrons at the maxima of the square of the wave function (Born maxima) highlights the rigidity of the same-spin electron blocks and validates the LDQ framework in atoms and molecules. A generalized LDQ model accounts for all bond types, including covalent, polar covalent, ionic, dative, and electron-deficient, and directly incorporates electron correlation effects, providing a rigorous yet intuitive approach to bonding. This perspective also reveals fundamental flaws in conventional mean-field descriptions that ignore the correlated motion of electrons. By bridging traditional and quantum paradigms, the generalized LDQ model offers a robust tool for understanding chemical bonding, with implications for education, experimental design, and theoretical advancements.

## Graphical abstract

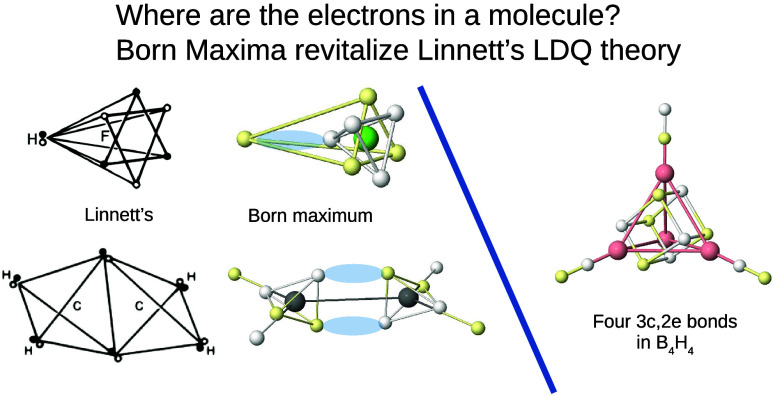

## 1 Introduction

It took 50 years since in 1866 Edward Frankland introduced the term *bond* in a paper published by the Journal of the Chemical Society,^1^ until Gilbert Newton Lewis presented his seminal work *The Atom and the Molecule* in 1916 and the electron pair came to life.^2^ By 1920, Langmuir had formalized much of Lewis’ ideas into a set of rules, coining everyday terms like the octet or the covalent bond. Since then, the elusive chemical bond has become the central concept of chemistry, and, today, almost one hundred years after quantum mechanics had provided solid roots to the pair through basic antisymmetry requirements, practicing chemists still push arrows. During their years of basic academic training, experimental chemists are exposed to a strange hodgepodge in which electrons or electron pairs are linked to rough quantum-mechanical objects, e.g., atomic and molecular orbitals endowed with their probabilistic interpretation, while they are simultaneously taught to manipulate them as pseudoclassical particles dwelling in real space. Although every chemist learns the foundations of quantum mechanics, few acquire the proficiency level required to escape the corset imposed by textbooks and monographs, dominated by the orbital (mean-field) paradigm. We all learn about the indistinguishability (equality) of electrons, but we are soon taught that, to paraphrase George Orwell, *some electrons are more equal than others*.^3^ In the end, chemists internalize how the Schrödinger equation is solved in the mean-field approximation, which provides one-electron states in the energy domain that are eigenstates of all the symmetry operators that commute with the Hamiltonian. In atoms, this leads to the *n*,*l*,*m*,*m*_*s*_ labeling and the shell and subshell structure that justifies the octet rule and the periodic behavior of the elements. In molecules this line of reasoning leads to all sorts of electron counting and symmetry rules, from Wades’,^4^ to Woodward–Hoffmann’s.^5^ Despite the approximate nature of the mean-field approximation, organic and inorganic chemists have developed solid *intuitions* that take them from the orbital domain and its symmetry labels, e.g., σ,π, to predictions. Many are aware that this view has its shortcomings, sometimes we even find in textbooks that two equivalent banana bonds can also describe the double bond in ethylene,^6^ but unorthodox thinking is rather effectively suppressed. Chemists, characterized by their pragmatism, easily adopt the shortest route to rationalize their experimental findings. If delocalized states suite their needs, like when trying to understand *aromaticity*, they use them. If not, they are not ashamed to lose their cherished symmetry labels and turn to localized orbitals instead, or even incorporate valence bond descriptions into their toolkit. It is the end that justifies the means.

The influence of the broad-brush quantum mechanical descriptions on contemporary chemical thinking was such that the octet rule became inextricably linked with the closed-shell nature of an s^2^p^6^ shell. At the same time, Lewis’s original suggestion of a cubical atom has been largely forgotten. Figure 1 reproduces how Lewis imagined the distribution of the valence electrons of second-row atoms, positioned at the corners of a cube. The eight electrons of Ne, not shown, would complete it. Atoms tended to complete the cube when forming molecules, leading to several possibilities that we call today ionic and covalent bonding. It is never too late to recognize the genius of Lewis, who even proposed that Coulomb’s law should fail at short distances well before the advent of modern quantum mechanics. After the quantum revolution, the electron pair survived as an opposite-spin couple of electrons, and so did the Lewis-Langmuir rules, but the cubical atom faded. Over the years, in parallel with the development of digital computers, the molecular orbital method of Mulliken and co-workers surpassed Pauling’s resonance theory in its ability to provide accurate calculations. Hence, the community adapted its thinking to the mean-field one-electron orbitals, which harbor two opposite spin electrons in closed-shell species.

**Figure 1:**
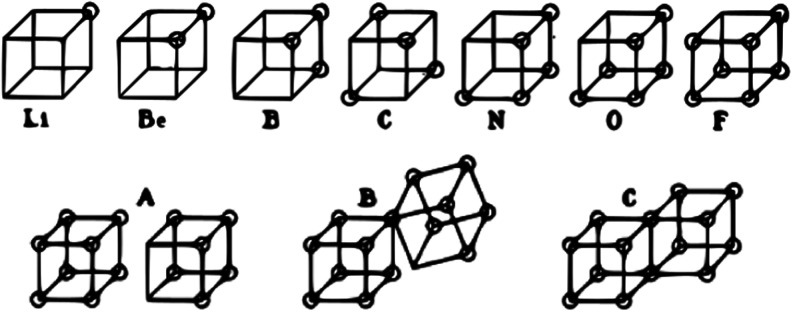
(Top) Lewis cubical atom description of elements Li through F. (Bottom) Ionic, partially shared, and fully shared representations of the I_2_ molecule. Reprinted with permission from ref 2. Copyright 1916 American Chemical Society.

Over the years, as computational chemistry evolved, tools were developed that more or less satisfied the chemists’ desire for Lewis pairs. For those less concerned with theoretical rigor, localized orbitals of one of the many existing flavors were sufficient. Others, insisting on the nonunique, approximate nature of these objects, developed techniques that focused on quantities that did not depend on orbitals (i.e., orbital-invariant descriptors). In these techniques, the electron density ρ played an important role, being experimentally accessible and providing the basis for density functional theory (DFT). Besides introducing an atomic partition of real space in the quantum theory of atoms in molecules (QTAIM),^7^ several scalar fields like the Laplacian of the density, ∇^2^ρ, or the electron localization function (ELF) of Becke and Edgecombe,^8^ were soon found to reveal the shell structure of atoms and how this partially or fully coalesces into electron pairs in molecules. Figure 2 shows localized orbitals, the four tetrahedrally distributed maxima of −∇^2^ρ around the O atom, and an isosurface of ELF in H_2_O. All can be used to recover four electron pairs or to support Gillespie’s valence shell electron pair repulsion (VSEPR) model.

**Figure 2:**
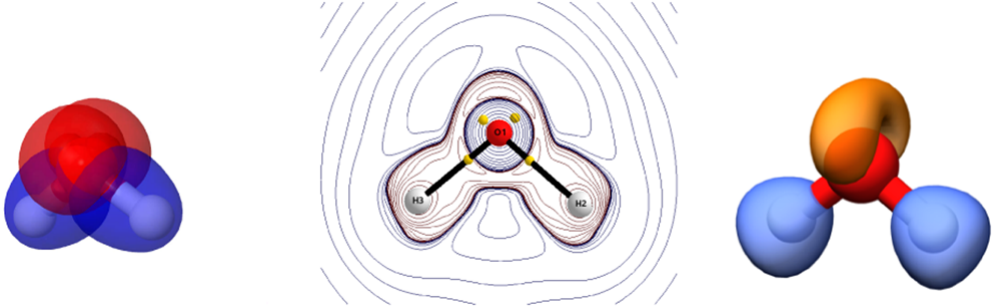
Three modern ways to access Lewis pairs exemplified in the B3LYP/cc-pVDZ H_2_O molecule: Valence Boys localized orbitals, with the rabbit lone pairs of oxygen colored in red (left). Isocontours of ∇^2^ρ (middle). Isosurfaces of ELF = 0.8 au (right).

Lewis did not know about electron spin when he proposed his cubical atom and struggled to fit radicals, ozone, and other simple molecules into his picture. However, after some preliminary studies in the 1950s about the role of spin in the spatial distribution of electrons in atoms, John Wilfred Linnett wrote a paper in Nature in 1960,^9^ later expanded in 1961,^10^ in which he proposed a modification of the Lewis-Langmuir octet rule. According to Linnett, the effect of spin was so important that the eight valence electrons of Neon should better be understood as two interpenetrated tetrahedra of opposite spin electrons, the so-called double quartet. Among the same spin electrons of each of these quartets acted extremely strong forces (Fermi correlation in today’s parlance), while between the two tetrahedra, only the weaker *charge correlation* (today’s Coulomb correlation) was operative. In the absence of other fields, the two quartets would minimize their interelectron repulsion by forming a perfect cube, as in the Ne atom. In molecules, the effect of the field induced by the extra positive centers would likely dominate over Coulomb’s correlation, and the two tetrahedra would easily rotate with respect to each other to maximize the number of vertices aligned with the new nuclei. This process leads to the formation of coincident corners allocating electron pairs. In the 1960s and early 1970s, Linnett and others showed how this simple, yet powerful modification of the octet rule could systematize many facts, solve inconsistencies in Lewis structures, and even eliminate the need for the concept of resonance. Figure 3 shows how easily the 1 + 3, 2 + 2, 3 + 1, and 4 + 0 Lewis+lone pairs of the HF, NH_3_, H_2_O, and CH_4_ molecules, respectively, appear according to these rules. In HF, for instance, the three lone pairs around the F atom would minimize Coulombic repulsions by a 60° rotation of the triangular faces of the up and down spin quartets, forming a star that would rotate freely around the internuclear axis. Linnett’s double quartet model (LDQ) was particularly successful in understanding multiple bonding. The single, double, and triple C–C bonds in ethane, ethylene, and acetylene appear when two tetrahedra centered around each of the C atoms share a vertex, an edge, or a face (see Figure 4). In the latter case, Linnet proposed again that the three bonding pairs would minimize repulsions as in HF, forming a freely rotating hexagon of alternating up-and-down spin electrons. LDQ was also very successful in providing a rationale for the triplet ground state of dioxygen: by decoupling the up and down spin quarters around each O atom, the majority spin tetrahedra shared a face, while the minority spin one shared just a corner. He also proposed structures for the singlet excited state of O_2_ and provided a simple rationalization for traditionally hypervalent systems, or a single structure for benzene (see Figure 4). All this was possible by expanding the 2c,2e bond to a more general 2c,ne link that was not easily accessible under the more restrictive Lewis-Langmuir rules. Despite the LDQ model’s connection to quantum mechanics and some attempts to apply it to other fields like transition metal chemistry by Luder,^11^ Linnett’s ideas did not last long,^12^ only surviving today in a handful of textbooks. Whatever the historical reason behind this, we will show that modern theoretical chemistry supports Linnett’s claims, with updates on concepts unknown in the 1960s.

**Figure 3:**
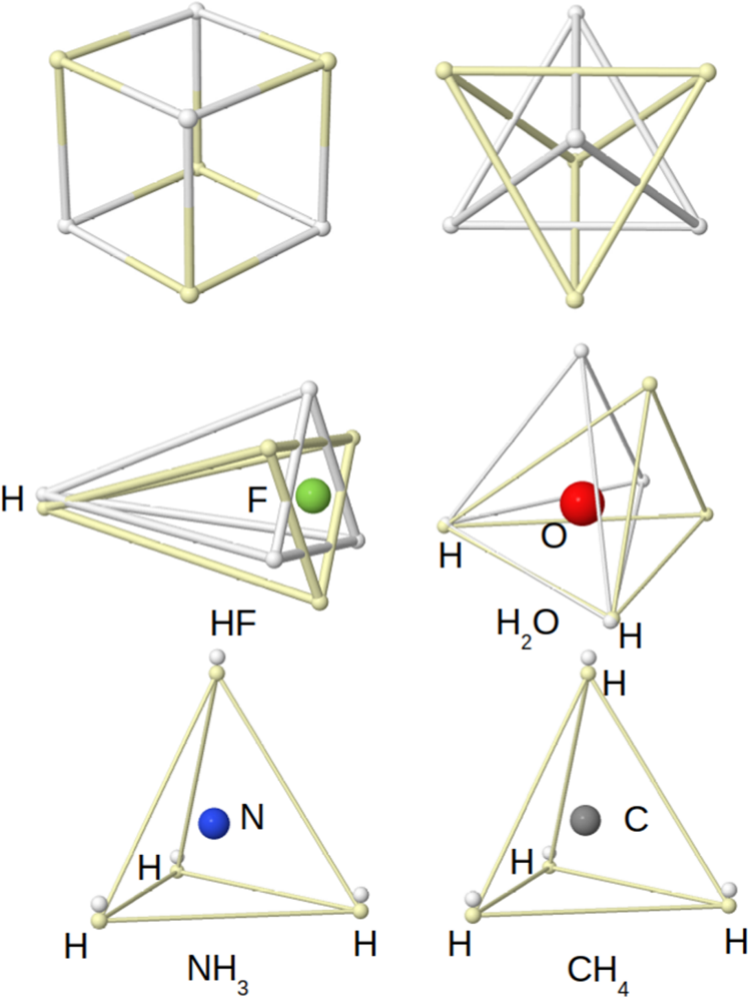
Linnett’s double quartets: Minimal repulsion cube (top), and coalescence into pairs (rest).Opposite spin electrons are depicted as white/yellow small spheres.

**Figure 4:**
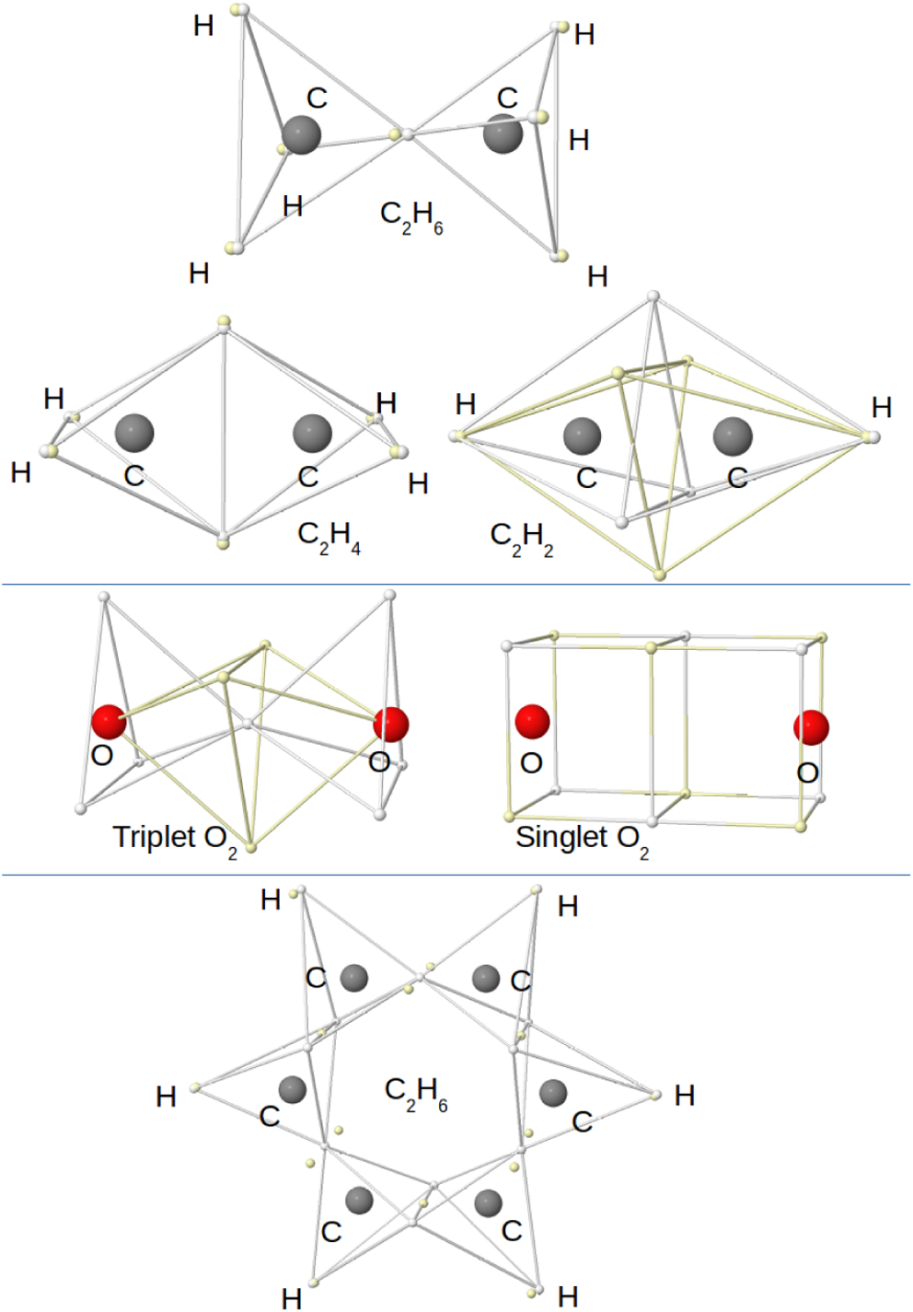
LDQ view of multiple C–C bonds (top), the triplet and first excited singlet state of dioxygen (middle), and benzene (bottom). Opposite spin electrons are depicted as white/yellow small spheres.

To do it, we just need to locate the positions of electrons in molecules. This is certainly possible in quantum mechanics (QM), for |Ψ­(***x***_1_,···, ***x***_*N*_)|^2^ provides the probability (density) to find the *N* electrons of a system at general positions in space (recall that ***x*** ≡ σ, ***r*** is a spin-spatial coordinate). Given the probabilistic nature of QM, the global (and local) maxima of |Ψ|^2^ are particularly relevant to us, indicating the most likely position of the electrons when we take, let us say, a *snapshot* of the system. Although this research program was well-known to early fathers of theoretical chemistry like Lennard-Jones,^13^ it also faded fast as the accuracy of calculations improved and the need for interpretations decreased, although the field was later revitalized after the work of Savin and Scemama,^14^ Lüchow,^15–17^ and Schmidt,^18,19^ among others. These works have shown that the so-called Born maxima provide vivid images of the electron distribution that are impressively close to Lewis’s dream, as beautifully shown by Heuer, Reuter and Lüchow, in ref 20., where dot Lewis structures are straightforwardly reconstructed from the Born maxima. Some of the systems considered here can also be found there.

We now show that these images are even closer to Linnett’s picture. After a few details on the computational setup we show how the distribution of electrons in atoms persists and can be easily interpreted in terms of generalized same-spin quartets that we call spin polytopes. With this tool we provide two general rules that generalize Linnett’s LDQ model, and then examine several exemplary chemical bonding types to show the power of the approach.

## 2 Computational Details

Equilibrium geometries of all the systems explored in this work have been obtained at the B3LYP/cc-pVDZ level of theory, except otherwise stated, with the GAMESS code.^21^ The 3*N*-dimensional maxima of |Ψ|^2^ have been obtained with our PROMOLDEN^22^ code from GAMESS complete active space (CASSCF) wave functions at the B3LYP geometry or from variational quantum Monte Carlo calculations through the AMOLQC suite.^23^ In this latter case, B3LYP single determinant pseudowave functions using the cc-pVDZ basis were obtained with GAMESS. In a second step, pure variational quantum Monte Carlo (VMC) calculations were performed and the coefficients of Jastrow factors to include the effect of electron correlation were optimized, such that Ψ = *e* ^*U*^Φ. *U* is expanded in terms of explicit interelectron and electron–nucleus coordinates. Details can be found in the Supporting Information (SI, section S1). During the VMC runs, a branch is taken to minimize–log­(|Ψ|^2^). This was done at equally spaced steps of the sampling procedure, using a combination of steepest descent and L-BFGS minimization algorithms.^23^ All second derivatives of the Born probability have been obtained with PROMOLDEN.

## 3 Generalizing Linnett’s Quartets: the Spatial Electron Distribution in Atoms

We begin by briefly reviewing the spatial electron distribution of ground state atoms, as revealed by the Born maxima, to show how modern theoretical results support Linnett’s insights, and where and how they diverge from them. Although some of this material is known and has been presented before,^24^ it is crucial to the rest of our discussion. As in previous contributions, we use a constructive approach and keep the technical details to a minimum. Atomic nuclei will be located at the origin of our electron coordinate reference system. Starting with the H atom, the ϕ_1*s*_ orbital displays its maximum probability at the nucleus. If the reader is not theoretically oriented, they should not confuse the shape of |ϕ_1*s*_(***r***)|^2^ with the common radial distribution function (≈4π*r* ^2^ϕ_1*s*_(*r*)^2^) used in textbooks. In the ground state 1s^2^ He atom, the mean-field (Hartree–Fock, HF) wave function is the |ϕ^1*s*^(1) ϕ̅^1*s*^(2)|Slater determinant (SD) (a bar implies a β or down-spin electron). Since spinorbitals of opposite-spin electrons are orthogonal, it is easy to show that |Ψ|^2^ factorizes under this approximation into the product of α (up) and β (down) probabilities, so that different spin electrons are statistically independent. In the He case, this leads to a Born maximum (BM) in which the two electrons lie at the nucleus. Due to the Coulombic cusp condition, this is maintained at any nonrelativistic level of theory. Pauli’s principle forces any new electron to lie outside the origin at the BM, but in any *Z* > 2 atom, two opposite-spin electrons, associated with the K shell, are always found at the nucleus. We will not consider these two core electrons anymore, and, from now on, electron positions will always be implied at the BM except otherwise stated.

We also build a polytope centered at the origin for each spin block. Thus, in Li, the third electron lies at a given distance from the nucleus, and in Be, the two valence electrons reside out of the origin at the same distance from it. At the HF level, the Be valence α electron can rotate freely with respect to the β one, and in doing so both can be located on top of each other without any change in probability. Coulomb correlation separates the two valence electrons, leading to a freely rotating valence dumbbell. The distances at which these electrons are located have been reported at several levels of theory and related to several shell structure descriptors, but are not relevant to our discussion.^25^

The valence of the boron atom in its ground state has two majority spin (α) and one minority spin β electrons. At the HF level, the *M*_*L*_ = 0, *M*_*S*_ = 1/2 component of the ^2^P state has two 2s2p_*z*_-like α electrons subject to an intense Fermi correlation that forbids them to occupy the same position and places them as far apart as possible at the BM, on the *z* axis. The remaining β electron (i.e., a 2s-like electron influenced slightly by its 1s partner) is transparent to the α block. This leads to an α polytope formed by two electrons lying on the *z* axis, occupying symmetric ± *z*_α_ positions. The β polytope is a single electron moving also freely on the surface of a sphere centered at the origin, with a slightly larger radius. If we correlate the two polytopes, we get a final triangular distribution, close to an equilateral triangle. Now the α dumbbell is not strictly centered at the nucleus. The different rigidity of the response of the Born probability to a perturbation of the position of same-spin electrons within a polytope, or opposite-spin electrons between the two different spin polytopes can be easily measured through the curvature of the *normal modes* of electron motion at the BM (i.e., the eigenvalues of the matrix of second derivatives of the potential with respect to the electron coordinates, see the SI, section S2). These eigenvalues can be obtained for several second derivative matrices: the (3*N* × 3*N*) α + β matrix for the full set of electrons, the 3*N*_α_ × 3*N*_α_ or 3*N*_β_ × 3*N*_β_ matrices for each of the spin blocks, or even the 3 × 3 matrix of a single specific electron. In B, and at the FCI/cc-pVDZ level of theory which we will use in the following, the lowest curvatures (eigenvalues) of the separated 3 × 3 β and 6 × 6 valence α blocks turn out to be 0.58 and 1.44 au, respectively. Notice that if core electrons are involved in the normal modes, the nuclear cusps will render these second derivatives meaningless, although if standard Gaussian basis sets are used the derivatives will still provide relevant information if the basis sets are not changed, *vide infra*)

Turning to the maximum *M*_*L*_,*M*_*S*_ component of the ^3^P state of the C atom, another α electron is added to the valence majority-spin block. A FCI/cc-pVDZ calculation leads to a BM where the four valence electrons form an equilateral triangular pyramid with a base of α electrons and a β electron at the apex. The lowest curvatures to deformation of the β electron and the α triangle, obtained after diagonalizing 3 × 3 β and 9 × 9 α valence second derivatives matrices, are 1.08 and 3.02 au, respectively. The maximum *M*_*S*_ component of the ^4^S state of N has 4 α electrons that, at the BM, form a tetrahedron. The β electron lies on top of the center of one of its triangular faces, and the lowest curvatures of the minority and majority spin polytopes are now 0.11 (this is a spherical S state) and 5.30 au, respectively. Notice how the stiffness of the majority spin polytope increases with the number of electrons and the nuclear charge, as expected. Turning to oxygen, since the majority spin block became saturated at nitrogen, the β polytope grows in electron population. At the BM, the α polytope is a tetrahedron, and the β one is a dumbbell intersecting one of the tetrahedron’s *C*_2*v*_ axes. The lowest curvatures of the minority and majority polytopes are now 5.00 and 7.04 au, respectively. In fluorine, the β polytope is a triangle rotated 60° with respect to one of the triangular faces of the α tetrahedron, and the curvatures change to 8.70 and 8.94 au, respectively. Upon closing the shell in Ne, the two polytopes become equivalent interpenetrated perfect tetrahedra forming a cube, exactly as proposed by Linnett. The two curvatures are now equal, peaking to 10.94 au. Notice that at the mean-field level, these two tetrahedra rotate freely with respect to each other, while at the correlated level, these rotations (3-fold degenerate) display a small curvature of 0.37 au. As already described,^15,25^ adding an extra electron starts a new shell. In Na, the new 3*s*-like electron is located at the BM at a larger distance from the nucleus, on top of the center of one of the faces of the cube formed by the eight electrons of the L shell (the L cube in the following). This is equivalent to Li if we ignore the L shell, and the M shell grows for third-period atoms following the same patterns just commented until two interpenetrated L and M cubes appear in Ar. Pictures of these arrangements can be found in the SI, section S3.

The above description discloses the physical basis of Linnett’s model and the rationale for its success. All that is needed is an image of electron shells lying in a narrow range of distances from the nucleus together with a crude notion of intense *Pauli repulsions* acting on same-spin electrons and weaker Coulombic interactions experienced by any pair of electrons. This leads to the following two rules, from higher to lower hierarchy: (1) each of the same-spin electron blocks of the valence of an atom adopts a very stiff configuration that minimizes the mutual Pauli repulsion of their electrons, (2) the two rigid opposite-spin polytopes rotate to minimize their Coulombic interactions. In the mean-field approximation, and for spherical states, the rotation of the two-polytopes is free. Coulomb correlation imposes some stiffness on these interblock motions, which however remain much more facile than their intrablock equivalents. A Linnett’s quartet has become a spin polytope in the general case.

The geometry of same-spin electron polytopes built from indistinguishable, equivalent electrons minimizes their *Pauli repulsions* by placing them at appropriate shell distances as far as possible from each other. This is basically J. J. Thomson’s problem^26^ of finding the minimum energy distribution of a set of equivalent charges on a spherical surface or, if we like, a restatement of the VSEPR model. Thus, using rule 1 the *n*-electron polytopes (with center of mass at the nucleus) would be a dumbbell, an equilateral triangle, a tetrahedron, a trigonal bipyramid, etc., for *n* = 2,3,4,5. Rule 2 rotates two of these rigid objects around the nucleus to minimize their Coulombic repulsions. These steps explain the BM of the ground state of atoms. As an example, in F we have *n* = 4,3 for the majority and minority spin blocks, respectively, and we need to rotate an α-tetrahedron around a β-triangle. This leads to the BM of the F atom. Rules 1 and 2 also explain why Linnett’s model is many times successful at explaining excited states. The excitation energy is usually unable to modify the rigid structure of the polytopes, but allows for higher-energy isomers when applying rule 2. For instance, in the *M*_*S*_ = 0 components of the first two excited state singlets of O (^1^D and ^1^S), two equivalent equilateral triangles for the up and down spin blocks should be expected. In the lowest-lying D state, the two triangles have their centers at the nucleus, but are contained in two orthogonal planes so that, for instance, one triangle lies on the *xz* plane with one electron on the positive *z* axis and the other triangle is on the *yz* plane, with one electron on the negative *z* axis. This minimizes repulsions in 3D. However, in the ^1^S state the disposition is planar, with a hexagon of alternating α and β electrons formed by two coplanar triangles of opposite spin electrons centered at the nucleus and rotated 60° with respect to each other (see the SI, section S3, for plots).

An alternative image, closer to the background of contemporary chemists uses *hybrid* or *equivalent* orbitals, introduced by Pauling in 1931,^27^ and further developed from a more rigorous point of view by Lennard-Jones and co-workers.^28,29^ In our context, we recall that any subset of same-spin spinorbitals of a Slater determinant (SD) can be subjected to a unitary (orthogonal if real-valued) transformation without altering the SD. Turning back as an example to a mean-field description of ^2^P-boron, the *M*_*S*_ = 1/2, *M*_*L*_ = 0 SD is |1s1̅s2s2̅s2p_*z*_|. Since two opposite spin electrons will lie at the nucleus at the BM, we exclude the 1s^2^ core and rotate the 2s2p_*z*_ α block to form two equivalent 2sp_1_, 2sp_2_ α functions that point in opposite directions along the *z* axis. We are thus led to two sp α hybrids that minimize repulsions (or that maximize the distance among the hybrid centroids) and one nonhybridized β s function. This immediately leads to the previously described mean-field BM by placing electrons at the probability maximum of the sp hybrids and the remaining β 2s function. Separate partial localization of the valence α and β orbitals for different electron counts lead, in the general case, to the well-known sp^*n*^ hybrids and the above-mentioned polytopes.

Localization provides another route to discover the shapes of *n* > 4 polytopes. For instance, the distribution of the 18*e* M shell of atoms (first encountered in Cu^+^ or Zn^2+^ as 3s^2^3p^6^3d^10^) corresponds to two equivalent interpenetrated 9*e* polytopes. We have previously described them^24,25^ in terms of Thomson’s polyhedra, but can equally be obtained by localization of a set of nine sp^3^d^5^ orbitals. This leads to a tricapped trigonal prism, experimentally realized, e.g., in ReH_9_^2–^, and to an 18*e* shell that can be described as three *hcp*-like hexagonal layers of alternate up and down electrons. Similarly, the 32*e* N shell is built from two interpenetrated 16*e* polytopes that can be visualized by localizing a sp^3^d^5^f^7^ shell. The final distribution is remarkably spherical, as seen in Figure 5. We will use white and yellow color to distinguish different spin electrons in all the following plots. These simple principles allow us to systematize more complex cases easily. For instance, the BM for the ground ^6^S state of Mn^2+^ (3s^2^3p^6^3d^5^) is built from interpenetrated 9*e* majority-spin and 4*e* minority-spin polytopes. In neutral Mn, another 4s^2^ dumbbell from the N shell is added at a larger distance from the nucleus. Subtler effects, like those coming from the existence of electronic dipoles at the BM that influence molecular geometries, have also been discussed.^24^

**Figure 5:**
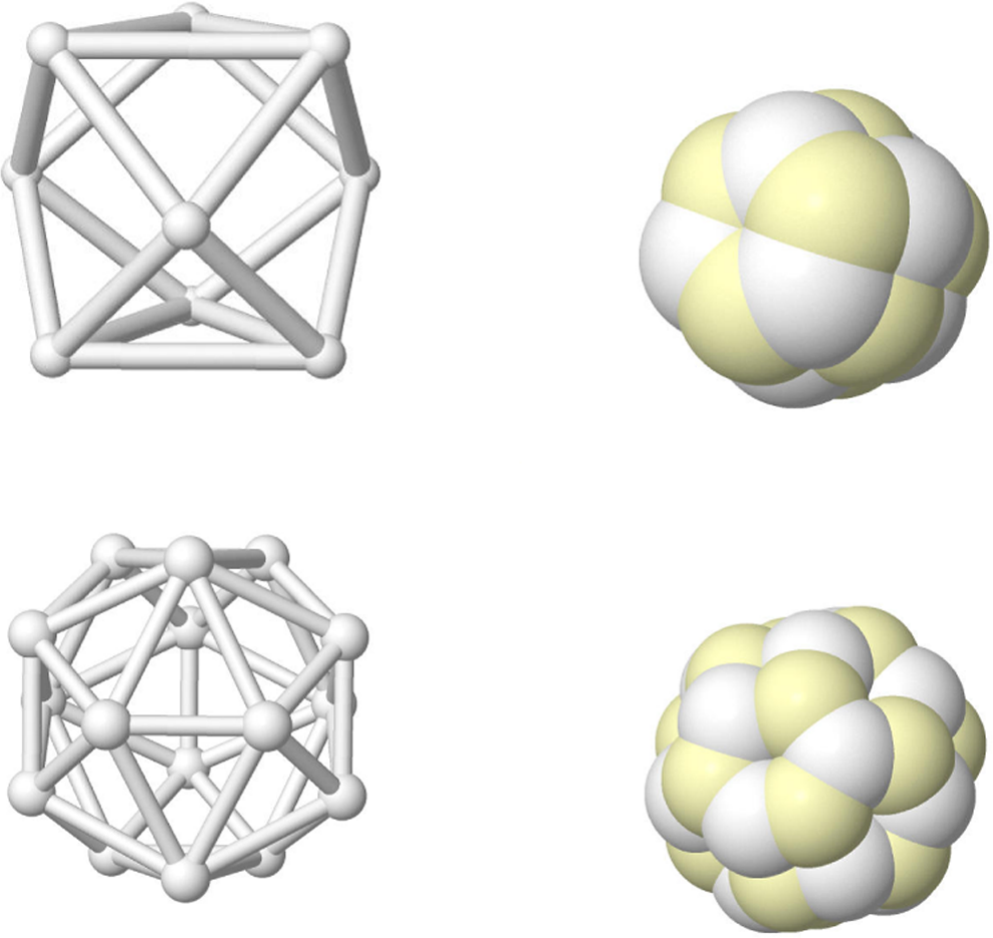
Same-spin polytopes (left) and total electron distribution (right) for the full M (top, 18*e*) and N (bottom, 32*e*) atomic shells.

## 4 Successes and Pitfalls in Molecules: Electron Localization and Delocalization, Persistence of the Shell Structure

Several features of the spatial electron distribution unknown to, or at least not taken into account by either Linnett or Luder are important for this modern revision of the LDQ model.

The first is related to electron delocalization, one of the deepest roots of chemical bonding. The presence of more than one nucleus in molecules implies that electrons can be found close to nuclei that differ from those we expect. In layman’s chemical language, bonding is due to orbital overlap. In VB parlance, we need to include both *covalent* and *ionic* structures. In terms of |Ψ|^2^, molecules are prone to display not just one global maximum, but a set of local maxima close in probability. For instance, in H_2_ the global BM has one electron on top of each H nucleus, as expected (there are two equivalent maxima in which the left and right nuclei bear up and down spin electrons or vice versa, but we are not dealing with spin and permutationally equivalent maxima). However, two other local maxima exist, in which the two electrons lie on either one or the other nucleus. The two sets are associated with the *covalent* and *ionic* VB structures. At the cc-pVDZ/FCI level, the |Ψ|^2^ probabilities of the covalent and ionic BMs are 0.022, and 0.008, respectively, with minimum curvatures to electron separation equal to 12.16 and 2.39 au. We recall here that since the electrons at the BM are located at nuclear positions the true second derivatives would the cusp condition be satisfied are not defined. In that case, first directional derivatives would provide similar information. However, if the basis set is not changed, as in the present calculations, the very different (wrong) curvatures still contain relevant information about how probabilities evolve under perturbations of the electronic positions. As the H–H distance increases the probability of the ionic maximum decreases so that at dissociation only the covalent one remains.

The second feature stems from ignoring the stiffness of atomic shells, namely, their persistence upon molecular formation. Many descriptors, starting with the Laplacian of the electron density, ∇^2^ρ, but also including others like the ELF or the electron localizability indicator (ELI)^30^ have revealed this rigidity over the years. Beyond relatively small expansions or contractions (typically smaller than 20%) due to the molecular environment, atomic shells persist in molecules.^24^ This impacts the images of a chemical bond as envisioned by Lewis and Linnett. Let us take the Linnett pictures of the HF and C_2_H_6_ molecules, depicted in Figures 3 and 4, as an example. The bonding pair is located close to the H atom, in HF, or in the middle of the C–C bond axis, in C_2_H_6_. This would imply either a very unlikely hydridic H atom, in the first case, or a distortion of the atomic shell of carbon much larger than what is actually found. As we are going to show, Linnett’s proposal of a rotation of the spin quartets to form Lewis pairs in molecules is correct, anchored in our rule 2, but the electrons of a given pair are still found at about the same distances from nuclei as in isolated atoms. This is nicely shown in the HF molecule.

Figure 6 displays three local maxima of |Ψ|^2^ in the hydrogen fluoride molecule. Their probability densities (top to bottom) are 4.03, 3.95, and 1.92 (×10^–4^) au, respectively. It is first clear that we can associate electrons with atoms. The first maximum is the global (*covalent*) BM, the second is another *covalent* maximum, and the third is an ionic F^–^ H^+^ structure. Ignoring the core electrons, and as predicted by Linnett, at the BM the eight valence electrons are distributed as two distorted spin quartets rotated such that their ternary axes align with the bond axis. However, the β tetrahedron (white spheres in the Figure) is considerably more distorted than the α, something that is easily rationalized in modern terms that Linnett and co-workers could not foresee. Considering the previously described stiffness of shells and taking an atomic viewpoint, the 4α (yellow) + 3β (white) polytope distribution of the isolated fluorine atom is notoriously conserved at the BM. We simply observe the same electron distribution as in a ^2^P isolated F atom (see the SI, section S3) such that the β spin tetrahedron is oriented toward the molecular axis. The H atom bears a single down-spin electron much as when it is isolated. This electron can be imagined to form a tetrahedron (quartet) with the three α (white) electrons at F. In this view, the bonding pair is formed by the two spin-coupled electrons on the axis, and the six electrons in the rear part of F that are placed on a ring-like hexagon orthogonal to the bond axis are linked to three lone pairs of F. Recall that the ELF of HF, for instance, shows such a lone pair torus around the intermolecular axis. All in all, the agreement with Linnett’s model is outstanding.

**Figure 6:**
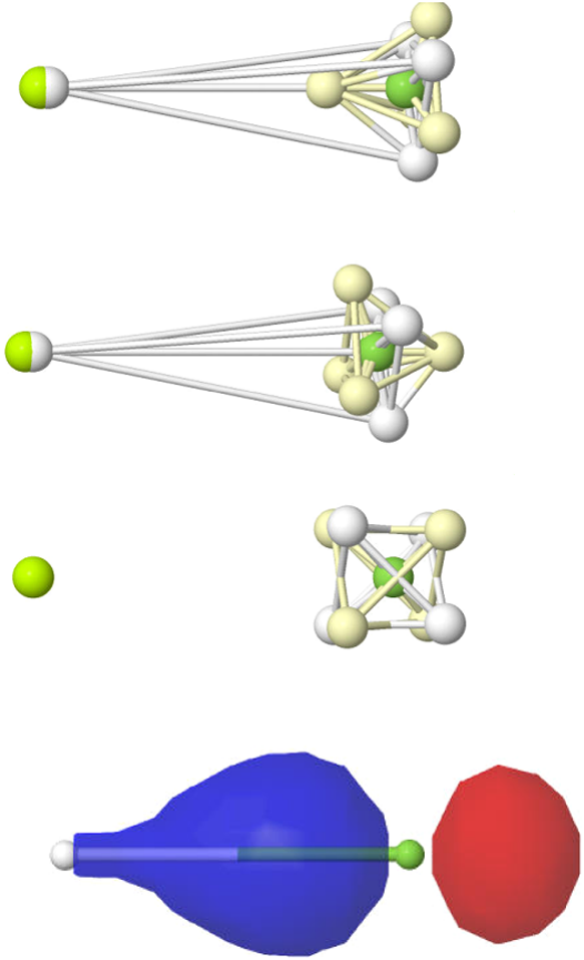
From top to bottom: Global and two local BMs in a frozen-core FCI/cc-pVDZ HF molecule. The position of the H and F nuclei are indicated by light-green spheres, and only one representative element of the rotationally equivalent maxima along the internuclear axis is shown. The bottom panel shows the |ϕ| = 0.39 au isosurface of the bonding σ natural orbital, with the F and H nuclei as small green and white spheres, respectively.

The second maximum is characterized by placing the F-like electron of the bonding pair also on the axis, but now in the rear part of the F atom. In molecular orbital (MO) parlance, this second maximum corresponds to placing one of the electrons of the bonding σ orbital, an H*s*F*p* hybrid with a large F*p* contribution, at the rear maximum of the *p* orbital (Figure 6, bottom). Finally, in the ionic maximum, the eight valence electrons are placed around the F atom, which behaves as a fluoride, thus displaying an electron distribution in which the L shell is filled and the two quartets form a (slightly distorted) cube. We stress how much of our standard chemical knowledge is contained in this type of analysis. Neutral or ionic *atomic species* are immediately recognized at the *covalent* and *ionic* Born maxima.

Readers can now complete the picture in CH_4_ in a back-of-the-envelope exercise as found in Figure 7. The C atom displays a tetrahedron of electrons around C, as in its isolated ground state, pointing toward each of the H atoms, which bear one electron. Along each bond direction a pair of opposite spin electrons, one at the H atom, the other much closer to C, at about the same distance as in the free atom L shell, can be associated with a Lewis pair. Notice that if the two electrons of each Lewis pair would be made to coincide in space, the two Linnett quartets would be placed on top of each other, as in the LDQ model. We have sketched that process by means of the arrows in the top part of the Figure. For instance, if the β electron at the nock of the vertical arrow pointing upward is made to coincide with its point, repeating this process with the three remaining arrows, Linnett’s CH_4_ in Figure 3 is recovered.

**Figure 7:**
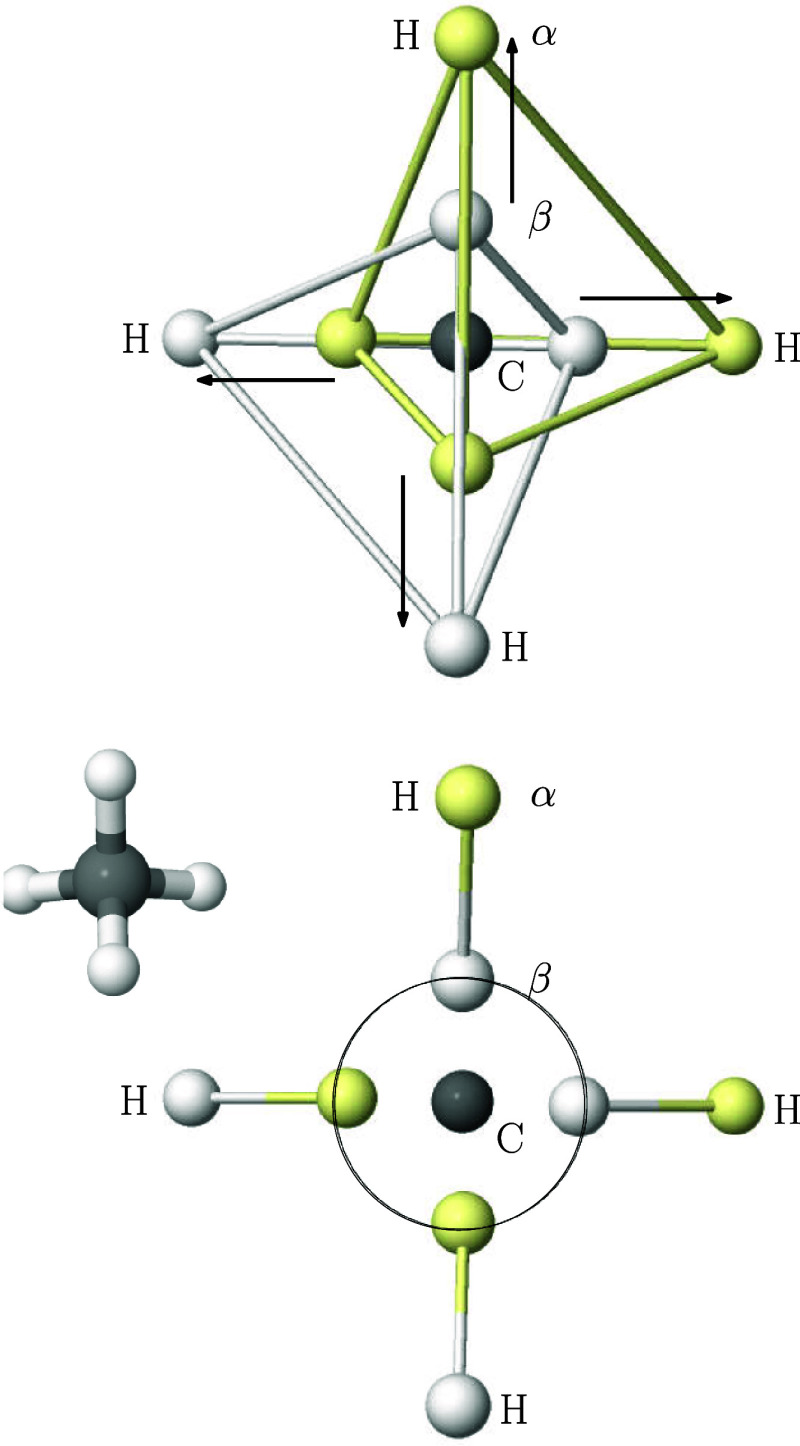
Global BM in CH_4_. Four electrons lie at the H atoms (external shell) and four closer to the C atom, at about the distance of its L shell (circle in the bottom panel). The two distorted Linnett quartets are shown in the top diagram, while the four Lewis pairs are highlighted on the bottom one. The perspective is orthogonal to one of the C_2v_ axes, as detailed in the small CH_4_ molecule on the top-left part of the bottom panel.

Similar principles apply in general. For instance, the triple bond in acetylene (Figure 8) is uncovered as three equivalent Lewis pairs formed from the juxtaposition of two triangular faces of the conserved L shell of each of the C atoms. The stiffness to rotation along the molecular axes of the two L tetrahedra is low, so other local maxima in which the tetrahedra are rotated 60 degrees, as in Figure 3 are also found. Again, if all the Lewis pairs would coalesce, Linnett’s picture would be exactly recovered. In benzene, Figure 9, the tetrahedral L shell around each C atom is clear, and there is one tilted Lewis pair between each pair of adjacent C atoms. Also, alternate up- and down-spin electrons that lie above and below the molecular plane are linked to the π clouds and are highlighted as triangles in the figure. Each L tetrahedron rotates rather easily with respect to its respective C–H axis so that the formal C–C bond order is 1.5. Except for the stiffness of the atomic shells that prevent the Lewis pairs from being located at the midbond positions, this is exactly the Linnett’s 2c,3e model of benzene.

**Figure 8:**
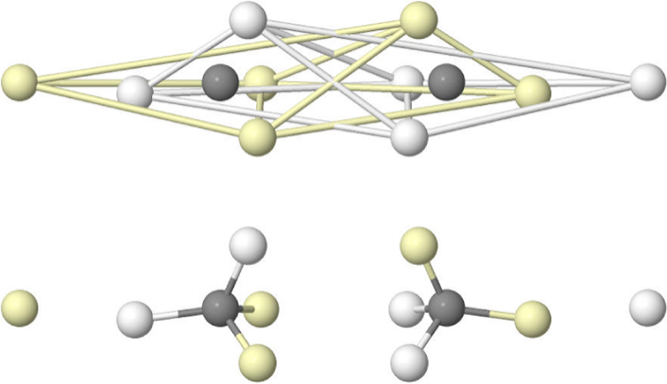
Global BM in C_2_H_2_. The Linnett quartets of acetylene are shown in the top diagram, while the conservation of the valence shell in the C atom is found in the bottom one.

**Figure 9:**
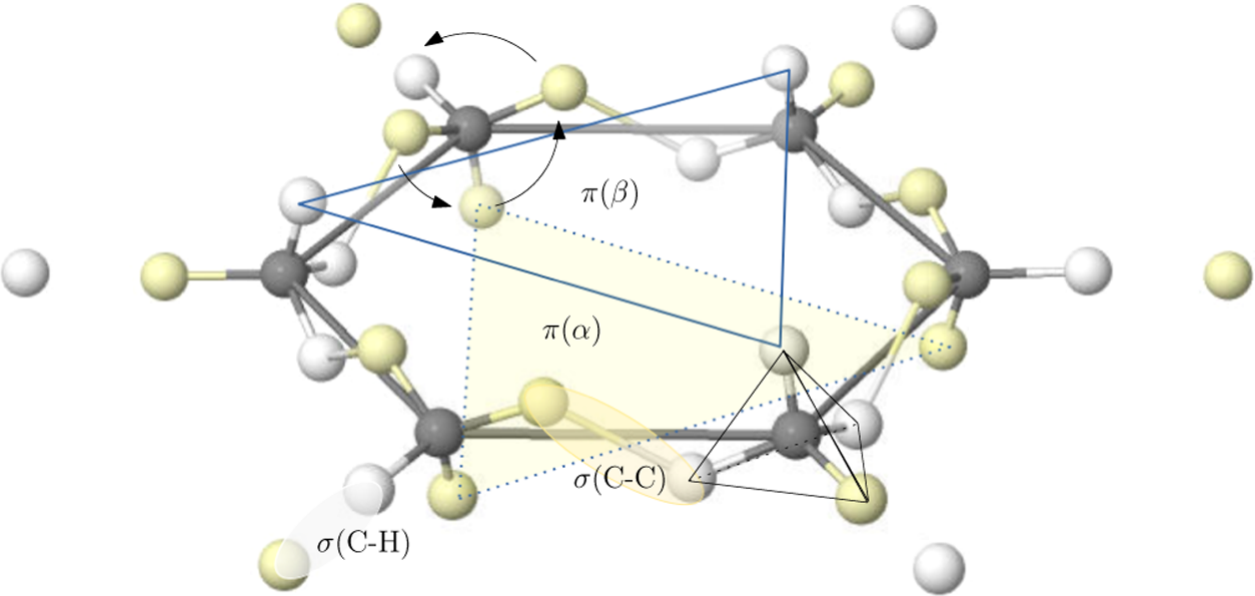
Global BM in benzene. Two different σ-like pairs are highlighted as colored ellipses and labeled. The six π-like electrons are formed by two equilateral triangles of α and β electrons below (dotted triangle) and above (solid triangle) the molecular plane, respectively. One of the tetrahedra of L-like valence electrons around the right-forefront carbon atom is shown, and the easy rotation of three C-associated electrons around one C–H axis is also sketched in the background-left C atom.

Summarizing, we firmly think that Linnett’s modification of the Lewis-Langmuir rules, revisited through the lens provided by the maxima of the square of the wave function, provides a valuable model deeply anchored in modern theoretical chemistry that can easily be understood and taught. Just a few modifications (our rules 1 and 2) of the original prescription are needed. We now turn to examine several bonding regimes in the light of these ideas.

## 5 Covalent Bonds

Shifting from the anomalous behavior of H_2_ caused by the absence of H cores, we now briefly examine how our rules provide very clear aufbau rules in standard covalent bonds

### 5.1 Single Bonds in Second-period Molecules

We have selected single X–X bonds from X = Li to F to exemplify the evolution of the spatial distribution of the electrons of a *Lewis pair* across a period. We have selected B3LYP/cc-pVDZ optimized geometries and computed CAS wave functions including all valence electrons and 12 orbitals for the Li_2_, linear Be_2_H_2_, planar B_2_H_4_, C_2_H_6_, N_2_H_4_, H_2_O_2_, and F_2_ molecules. Their BMs are pictured in Figure 10.

**Figure 10:**
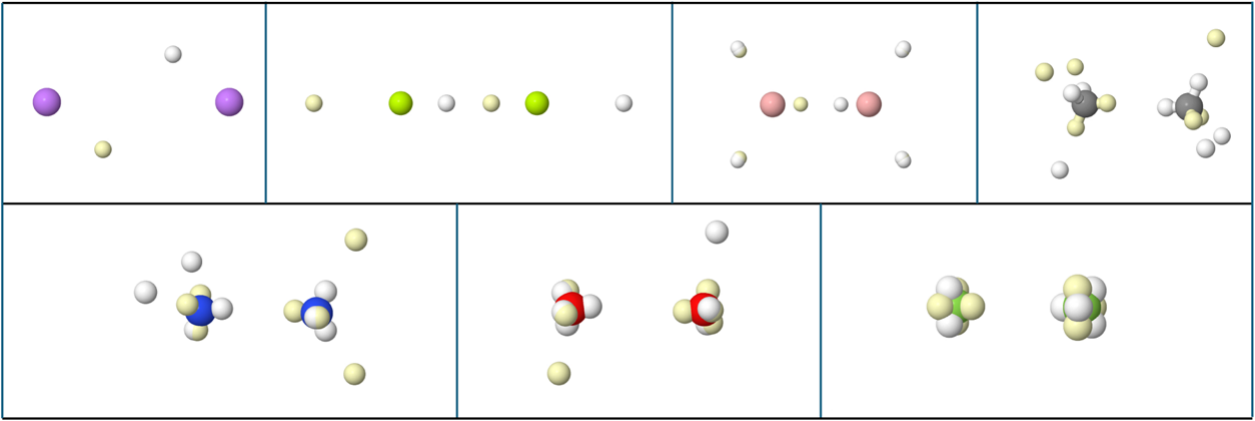
From left to right, top to bottom. BMs as obtained from CAS calculations for the single X–X bonds in the Li_2_, linear Be_2_H_2_, B_2_H_4_, C_2_H_6_, N_2_H_4_, H_2_O_2_, and F_2_ molecules. In Be_2_H_2_ and B_2_H_4_ each of the H nuclei bears two opposite spin electrons.

The two valence electrons of the Li_2_ molecule are located at the BM outside the bond axis, forming a soft dumbbell centered at the internuclear midpoint, with a lowest curvature to deformation of about 0.2 au. At the HF level, the two electrons lie exactly at the axis midpoint, corresponding to Linnett’s model. This disposition justifies the presence of a well-known non-nuclear attractor (NNA) of the electron density.^31^ We note that an unstable saddle point of |Ψ|^2^ also exists where the two electrons lie on the axis, each lying 0.14 Å closer to the nucleus than at the global off-axis maximum. This points to a release of excessive Coulomb repulsion as the reason behind the off-axis distortion of the global BM and provides an easily understandable explanation of why the first lowly occupied natural orbitals of dilithium are of π nature (see below for more information about non-nuclear attractors). It can be shown that, upon stretching the molecule, the angle between the dumbbell and the internuclear axis decreases until the two electrons collapse onto the axis. The electron density of Be_2_H_2_ displays two NNAs on the internuclear axis, 0.14 au to the left and the right of its midpoint. At the BM, one of the two valence electrons of each Be atom lies at its closest H nucleus, so the Be–H link is ionic-like in this sense (see below for more details). The other two valence electrons lie on the Be–Be axis, 0.65 au away from its center, forming an opposite spin Lewis pair. The global (all electron) normal mode of the probability Hessian that corresponds to a symmetric stretching mode of these two electrons has a small curvature of 0.83 au. A Boys orbital localization, for instance, provides two Be–H bonding hybrids heavily polarized toward the H atom, and a σ Be–Be localized orbital. An analysis of the total electron density and its localized orbital contributions is rather revealing. Along the Be–H line, both −∇^2^ρ and the density of the Be–H hybrid localized orbital display maxima only at the nuclei. However, along the Be–Be line, −∇^2^ρ has two extra noncore maxima, as shown in Figure 11, and the density of the Be–Be σ orbital displays also a central maximum. Considering that the Be–Be and Be–H internuclear regions are thus dominated by a single localized orbital contribution, the central maximum of the Be–Be localized orbital justifies the two electrons of its pair lying along the Be–Be internuclear axis, separated by Coulomb correlation. Similarly, the absence of a maximum along the B–H line forces the electrons of the pair to choose the H nuclear position, since the Be nucleus is already occupied by the 1s^2^ core at the BM. This simple argument rationalizes the *ionic* and *covalent* character of the BM in the two cases. As we will show, this behavior is rather general. In B_2_H_4_ the situation is analogous to Be_2_H_2_: the B–H links are ionic at the BM, while the B–B bond displays a Lewis pair with electrons that are closer and closer to the nuclei (0.96 au in this case) as *Z* increases and the L atomic shell shrinks. Again, both the Laplacian and the maxima of the square of the localized bonds allow us to tell the type of electronic distribution found at the BM. Notice that boron is still less electronegative than H, so a change is expected in carbon. Although Linnett’s quartets do not exist yet in any of these electron-deficient compounds, the general spin polytopes introduced before persist.

**Figure 11:**
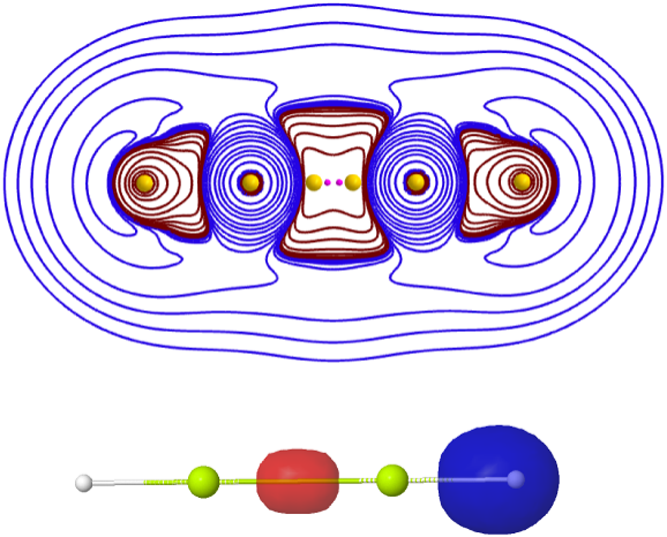
Isocontour map (top) of −∇^2^ρ for Be_2_H_2_ in a plane containing the nuclei at the CAS level. Positive and negative values are colored in brownish-red and blue, respectively. The positions of the maxima of −∇^2^ρ are marked with yellow spheres, with two non-nuclear maxima close to the center of the molecule as small magenta spheres. Isosurfaces (|ϕ| = 0.17 au) of the Be–Be (red) and Be–H (blue) Boys localized σ orbitals are found in the bottom panel.

The BM in ethane is bluntly covalent-like both along the C–H and C–C directions, and each C atom has four Lewis pairs around it. Should their position coalesce at the H nucleus or the C–C midpoint we would come to Linnett’s model. Since the atomic structure persists, we distinguish four valence electrons around C, at 0.72 au from it along the C–H directions, and 0.63 au in the case of the C–C bond. In the isolated C atom this is about 0.65 au at the same level of theory, so the expansion of the shell is larger in the slightly polar C–H bond. Now, both −∇^2^ρ and the square of localized orbitals show noncore maxima around the C atom in all cases. Two generalized Linnett quartets have appeared that are stable as we evolve to N_2_H_4_, H_2_O_2_, and F_2_. Along this series, we see (i) how the atomic shell structure of the non-H atoms (X) persist and (ii) how the distance from each of the two electrons of the X–X Lewis pair to its closest X nucleus decreases, from 0.51 to 0.40 to 0.35 au, respectively, on going from X = N to O, and finally F. The rotation of the quartets generates one, two, and three lone pairs. The alignment of these lone pairs with H atoms located at the other X partner is notorious in hydrazine and hydrogen peroxide. It can be used to justify their twisted geometries.

### 5.2 Some Homopolar Second-period Molecules

Since the BMs of second-period homodiatomics have already been considered,^15^ we only pay attention to a couple of interesting examples relevant to the LDQ theory. Provided that ^3^Σ_*g*_^–^ diboron and ^1^Σ_*g*_^+^ dicarbon possess six and eight valence electrons, respectively, not only the persistence of the electronic structure around each nucleus but also the global arrangement of all the valence electrons in the system deserve to be examined in the light of our rules. The BMs from B3LYP/cc-pVDZ optimized geometries and CAS wave functions, including all valence electrons and 12 orbitals, are represented in Figure 12. The left panels show how, in both cases, we neatly distinguish two ground-state atoms in the final molecule. In B_2_ it is two ^2^P B atoms coupled to a triplet, while in C_2_ two ^3^P carbons couple to a singlet. Notice the orthogonal arrangement of the two planes containing three electrons in each of the B atoms. Recalling our previous paragraphs on separate-spin orbital localization of the α,β-blocks, in a single determinant approximation the valence 2σ_*g*_ 2 σ̅_*g*_2σ_*u*_ 2 σ̅_*u*_ 1π_*u*,*x*_ 1π_*u*,*y*_ MOs can also be subjected to such a process. This leads, eventually, to two localized β functions of σ symmetry and four localized α functions that organize as a distorted tetrahedron. This can be used to understand the B_2_ BM, in which the total set of α valence electrons try to stay away as much as possible, given the nuclear constraints, by forming a distorted tetrahedron. Similarly, the β block provides a dumbbell along the internuclear axis. Of utmost interest is a united atom analogy, sketched in the right panels of the Figure, for the global disposition of the six valence electrons is just a distorted version of the polytopes found in the ground state of the oxygen atom, with also six valence electrons and which is, again, a triplet.

**Figure 12:**
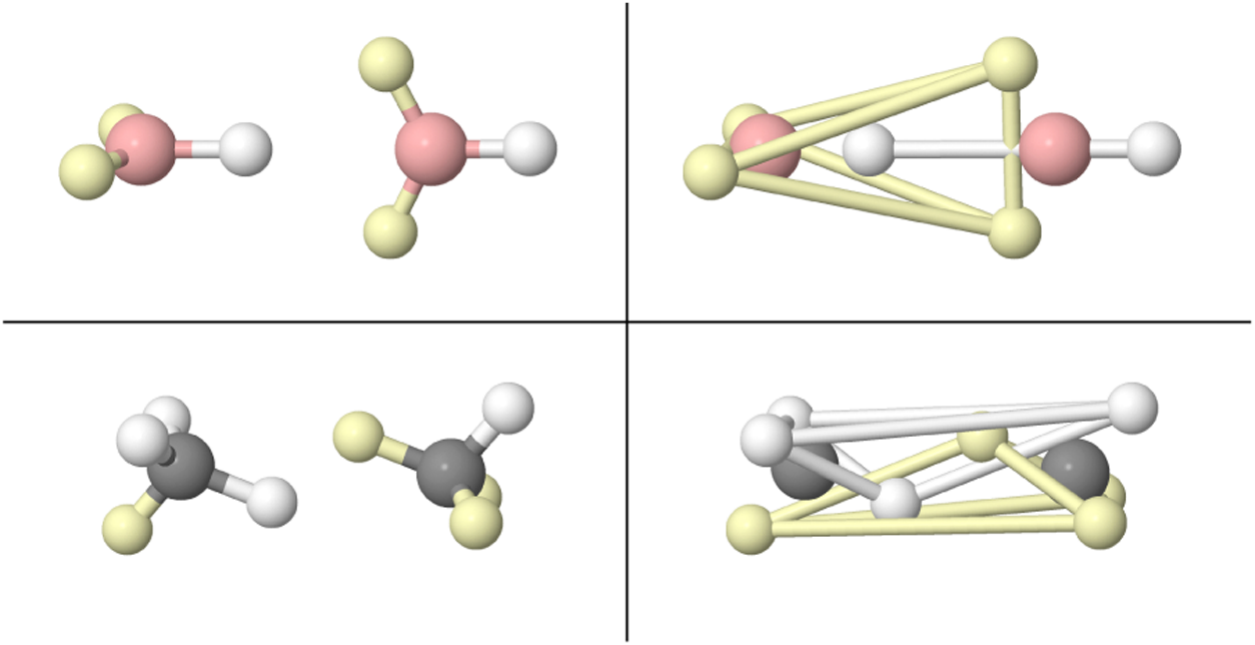
CAS BMs in B_2_ (top) and C_2_ (bottom). The left panels stress the persistence of the atomic structure, while the right ones focus on the global disposition of the valence electrons.

Turning to dicarbon, the united atom analogy would approach us to an eight valence electrons atom, Ne, and a singlet ground state with a (distorted) cubic arrangement. This is exactly what is found in Figure 12. As we increase the number of electrons in going to N_2_, O_2_, or F_2_, the united atom arrangement loses sense, for it would imply populating new atomic shells in contradiction with the persistence of the atomic structure. Their ground states can be easily understood as the coupling of atoms in either their ground or easily accessible excited states, and will not be further considered.

## 6 Ionic, Polar-covalent, and Dative Bonds

As the electronegativity difference of the atoms involved in a given chemical bond increases, the rules of the generalized LDQ model still apply after including the ground state (sometimes the low-lying excited states) of singly or multiply charged cations or anions. In Figure 13 we plot several pairs of alkaline halides and alkaline-earth oxides as examples: NaF, NaCl, KF, KCl, MgO, and CaO. In all cases, the ionization of the atoms is as expected from their formal oxidation states: singly ionized ions in the alkali halides, e.g., Na^+^ F^–^, and doubly ionized ones in the alkaline-earth oxides, e.g., Mg^2+^ O^2–^. This way, the BM in the top-left NaF molecule contains two well-isolated isoelectronic Ne-like ions that reveal the larger size of the L shell of the fluoride (or the smaller size of Na^+^). The evolution of size differences is apparent in all six cases.

**Figure 13:**
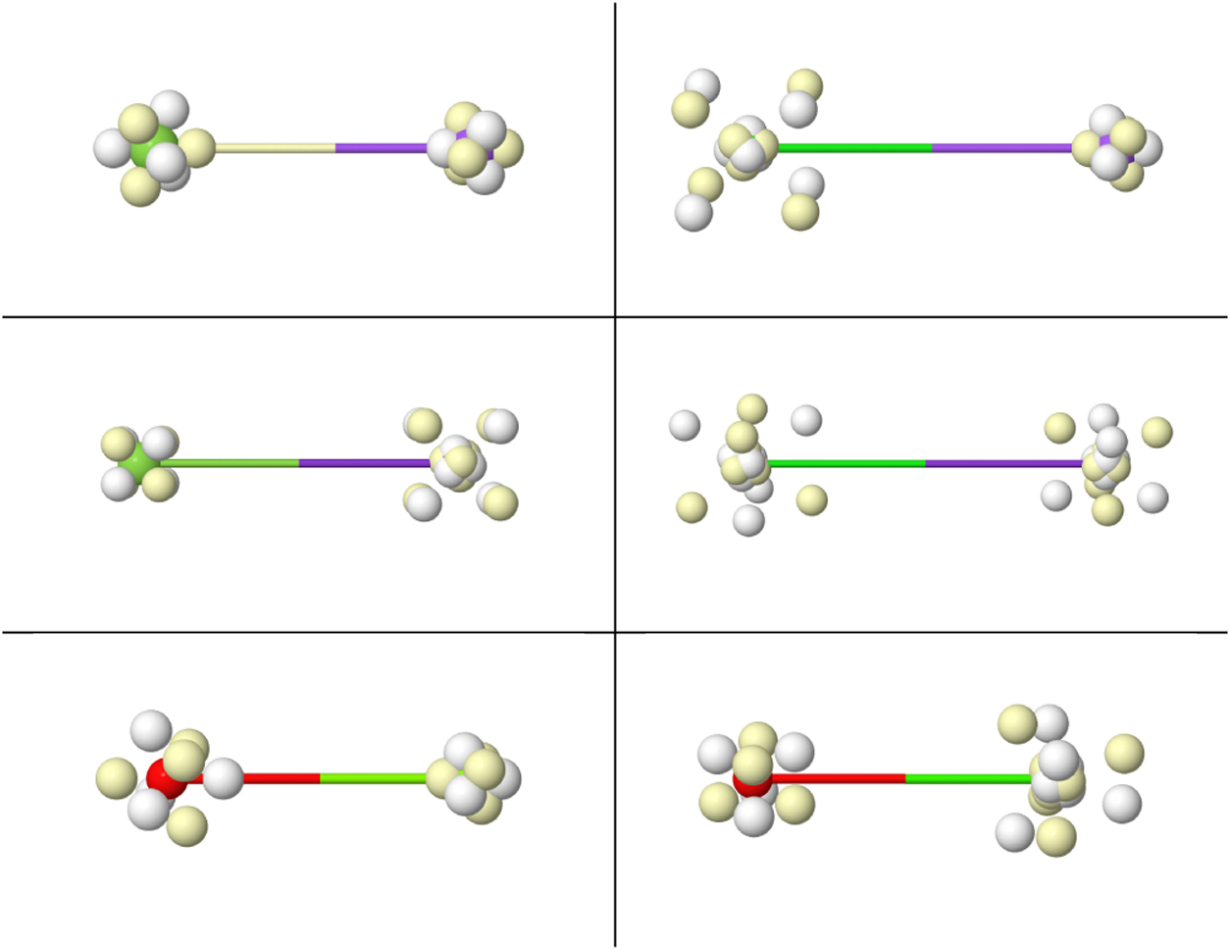
VMC BMs in several ionic diatomics. From left to right, top to bottom: NaF, NaCl, KF, KCl, MgO, CaO. In every case, the cation lies rightmost.

There are several points to consider. First, there is an exquisitely fine structure in the distribution of electron spins at the maxima. Within a single atom-in-the-molecule, e.g., in the Cl^–^ anion in NaCl, the inner L cube polarizes to minimize Pauli and Coulombic repulsions with the outer M cube. This core polarization propagates to reduced-dimensionality scalar fields like ∇^2^ρ and the ELF, although we will not consider this effect here. The fine structure also leads to spin opposition between electrons at different atomic centers. The two closest faces of the K and F cubes in KF are fully spin-coupled. Second, there are several modes in which the valence cubes of the cation and anion present to each other. Each cube can align a corner (C), face (F), or edge (E) along the internuclear axis. We distinguish in the Figure CC, FC, FF, and EE distributions. They differ in the ratio of Pauli repulsion to electrostatic attraction or, using another language, in the amount of σ and π bonding contributions. It will be worth investigating all these links in future works. Compact molecules with hard ions, like NaF or MgO, tend to favor CC distributions with dominating sigma interactions, while larger molecules with softer ions, like KCl or CaO display preferably EE alignments.

Finally, as the polarizability of the ions increases the BMs show a tendency toward distortion of the spin polytopes (in this case, tetrahedra). This can already be sensed in the K^+^ cation in KCl, where the cube’s face in front of the fluoride is slightly expanded with respect to the opposite face. This is even clearer in the FF CaO case.

The casuistry in polar bonds is extremely rich, but once the atoms-in-the-molecules of a given system are isolated one can easily interpret the global or local BMs in terms of the rules already presented. Figure 14 displays the CO, CO_2_, and NO_3_^–^ molecules as examples. CO displays a C atom with a very clear lone pair in the rear part of the internuclear axis and a B-like triangular electron distribution. This atom is coupled to an oxygen whose valence electrons can be viewed forming either a distorted cube with a corner that corresponds to the C α electron located on the axis or a distorted F-like seven-electron atom. One of the electrons of the lone pair is taken by the second oxygen in CO_2_, leaving a Be-like C atom and two oxygens similar to those seen in CO. Notice that if we build distorted cubes around each oxygen, the standard +4 and −2 oxidation states of C and O are recovered.

**Figure 14:**
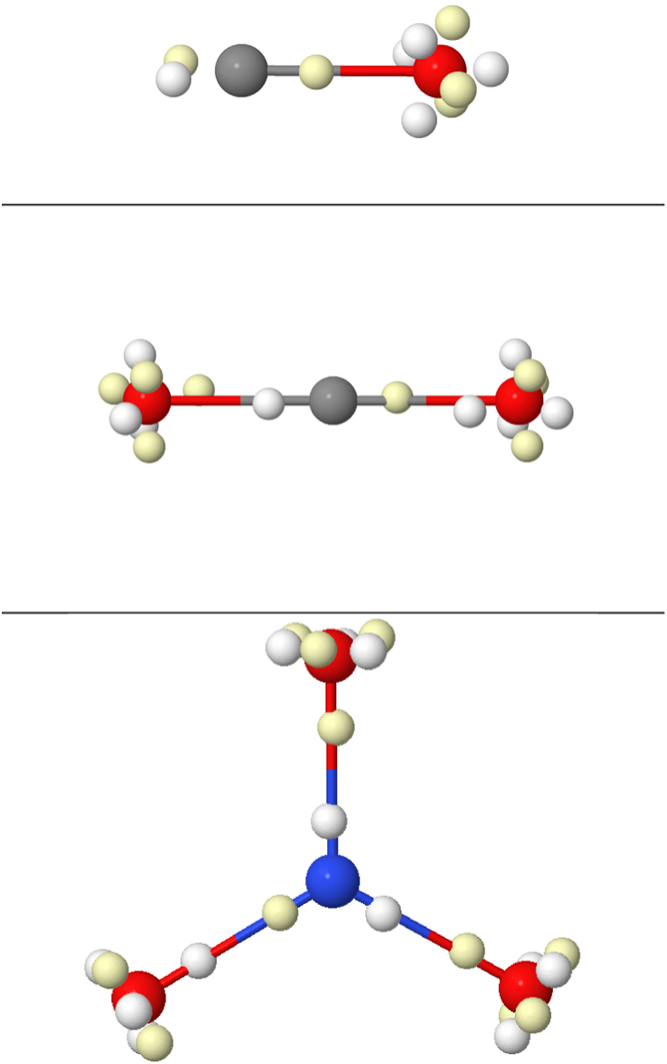
VMC BMs in CO (top), CO_2_ (middle), and NO_3_^–^ (bottom).

Since N is more electronegative than C, the electron distribution in the nitrate anion is neatly described as a B-like N^2+^ surrounded by three equivalent F-like O^–^ anions, with standard Lewis pairs along each NO axis. These two examples demonstrate how in the case of C–O bonds the oxygen atom tends to an oxide that is not fully achieved.

Dative bonds, which were mostly abandoned for decades, have been revitalized after the work of Haaland.^32^ Figure 15 shows the BMs of LiNH_3_^+^, SF6 and C_3_O_2_. The first system is similar to ammonia borane, and its BM shows a Li^+^ cation and a mostly undistorted ammonia molecule with its lone pair facing the lithium atom. The purpose of bringing SF_6_ to the fore here is 2-fold. First, the BM is blatantly ionic, so no octet expansion needs to be invoked. This agrees with the closed-shell nature of the S–F interactions revealed by ∇^2^ρ or the ELF. Other local Born maxima not shown display a larger number of valence electrons around the sulfur atom, without any octet expansion. For instance, one of the F atoms in these local maxima may display a neutral F distribution, with 7 electrons around it as in the top panel of Figure 6. The extra electron lies along the S–F line closer to the S atom. However, the probability of multiple neutral F atoms around the sulfur decreases very fast, and no local maximum with more than eight valence electrons around the S atom has been found. Regarding the aim of this subsection, we notice how the cubic L shells of each fluoride are oriented such as to suggest dative bonds to the S^6+^ central core. We stress how powerful the avoidance of Pauli repulsion can be, since along the F–F second neighbor directions the faces of adjacent cubes avoid same-spin contacts, forming a maximally separated network of spin-blocks. This face opposition is incompatible with O_*h*_ symmetry, so a certain type of frustration of the network appears (see below). Overall, this structure contributes to secondary fluoride-fluoride interactions that are easily measured through, e.g., the delocalization index.^33^ Anion-anion interactions are ubiquitous in ionic solids and have been the subject of intense debates. This type of analysis provides further hints about their nature.

**Figure 15:**
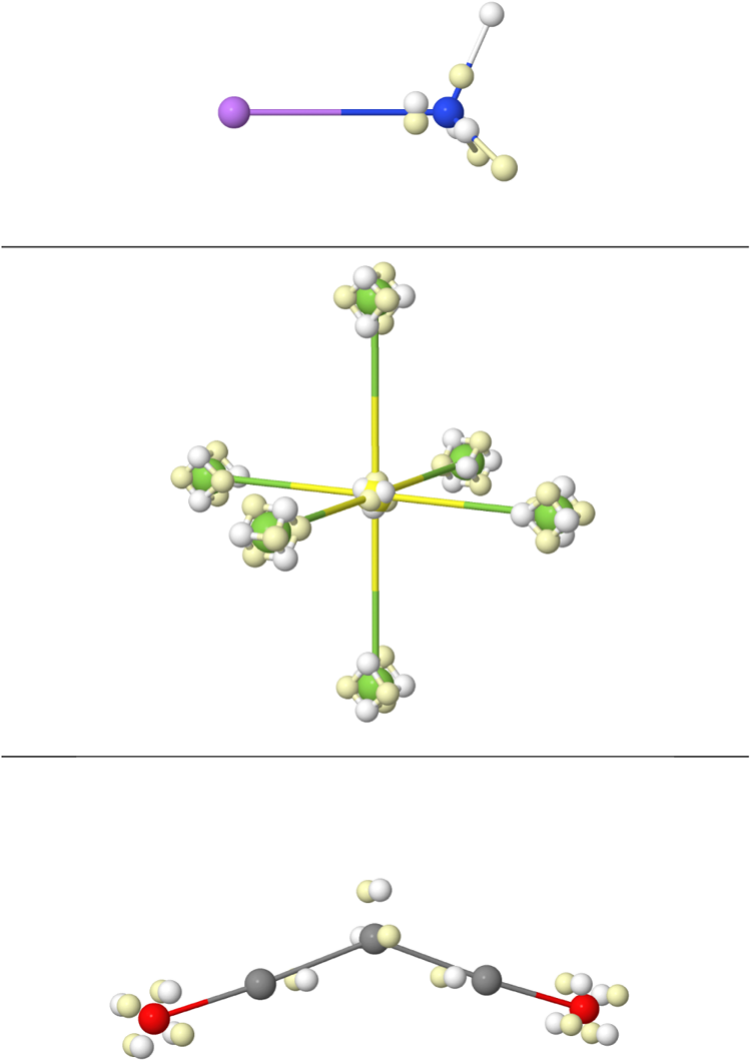
VMC BMs in LiNH_3_^+^ (top), SF_6_ (middle), and C_3_O_2_ (bottom).

Another interesting case is carbon suboxide, which displays an angular geometry understood^34^ as a zerovalent central carbon that bears two lone pairs (LPs), a so-called carbone. The carbonyl groups are sigma donors engaged in dative bonds with the central C, and the geometry is bent as a consequence of the lone pairs of the latter. Notice how the BM is directly read in those terms. The COs form dative bonds to the central carbon, which displays a planar distribution of its four valence electrons, corresponding to its ^1^D excited state.

## 7 Electron Deficient Systems and Electronic Frustration

Electron-deficient molecules have fascinated chemists for decades, leading, among many other insights, to the rich geometrical landscape (and chemistry) of boron compounds or the nowadays familiar concept of multicenter two-electron bonds. From an electron counting perspective, and in correspondence with many phenomena in spin physics, electron deficiency implies the lack of enough valence electron pairs to form as many bonds as those expected from the Lewis paradigm, leading to geometrical frustration of the Lewis pairs. As we will show below, the generalized LDQ framework provides an image in which this geometrical frustration of the electron distribution is key to understanding electron-deficient systems while preserving the local electron distribution around each atomic center. Provided that we aim to show how modern electronic structure theory abides by the generalized framework proposed here, we consider a few examples.

Li_3_^+^ is the first representative of a family of electron-deficient polyatomic lithium clusters. It exhibits a non-nuclear maximum of the electron density at the center of the equilateral triangle. Since there are two valence electrons for three nominal “bonds”, this system can be understood as an archetype of the 3c,2e bond (the simpler H_3_^+^ cation lacks cores and does not lead to a general family of compounds, although it would also serve our purposes). Figure 16 shows one of the three geometrical possibilities in the BM of this system (six if we take into account spin flips, see below). The two electrons lie on the molecular plane, parallel to one side of the triangle, at an equal distance from each of the Li atoms that flank the side. There is another maximum with the spins flipped. Overall, three arrangements and six spin possibilities are envisaged. Each electron can be associated with one of the Li atoms (as shown in the Figure). Thus, frustration and persistence of the atomic electronic structure define the 3c,2e bond. Let us notice that at the simple MO level, the doubly occupied valence σ_*g*_ orbital is a structureless object that extends over the whole molecule, and that this is the paradigm of a σ-aromatic molecule. We believe that the relationship between aromaticity, resonance theory, and geometric frustration deserves further study.

**Figure 16:**
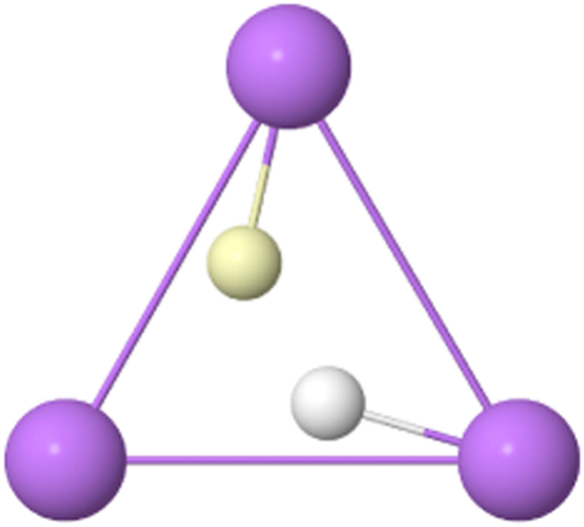
VMC BMs in Li_3_^+^. All the nuclei and electrons lie in the same plane.

Armed with these ideas, Figure 17 displays one of the many degenerate BMs found in the tetrahedral B_4_H_4_ molecule. Besides four standard B–H σ bonds, the cohesion of the B_4_ tetrahedron is understood in terms of four standard 3c,2e bonds. The left panel reveals how each B atom has a mostly planar triangular distribution of electrons as in the isolated ^2^P state of neutral B. One of these three electrons points to the H atom, while the other two are placed on two different faces of the tetrahedron, at a distance above the plane of the faces. The same frustrated pattern of the 3c,2e bond commented in Li_3_^+^ is patent, in agreement with the typical position of the maxima of localized orbitals representing the 3c,2e links. These are represented in the right part of the Figure, and have been obtained through our real-space natural adaptive partitioning method (rs-AdNDP),^35^ that provides an orbital invariant analogue of Boldyrev’s AdNDP procedure.^36^ Notice also that in a topological analysis of the electron density, the position of the ring critical points associated with each of the four triangular B–B–B faces lies out of the B–B–B planes, close to the maxima of the four 3c,2e localized orbitals, see the red representative in the Figure.

**Figure 17:**
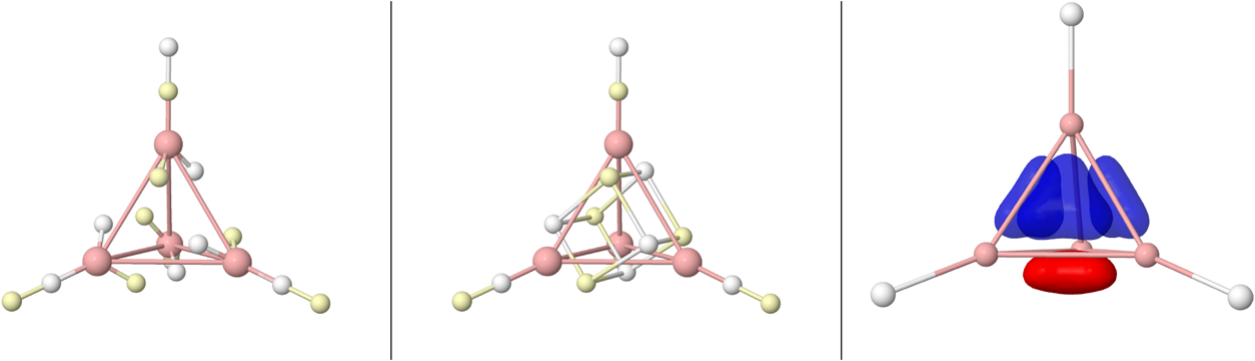
VMC BMs in B_4_H_4_. The left view emphasizes the atomic nature of the electron distribution around each B atom, the middle panel shows the cubic structure of the electrons engaged in 3c,2e bonds, and the right panel displays isosurfaces (|ϕ| = 0.23 au) of the four 3c,2e rs-AdNDP orbitals, obtained from the optimized B3LYP Kohn–Sham determinant. One orbital is marked in red to show how its maximum is located outside the plane of the three B atoms involved in the 3c,2e bond.

A closer look at the BM (central panel) reveals a fine structure hidden in the conventional one-electron MO picture: the eight electrons of the four 3c,2e bonds are intensely correlated. Once this is considered, it is unsurprising that they organize themselves into a cube of interpenetrated opposite-spin tetrahedra. Actually, all the possible configurations of the set of 3c,2e bonds can be obtained by concerted rotations of the two electrons provided by each B atom around their respective B–H axes, i.e., by rotations of the cube. Another united atom analogy is also possible, since the set of four 3c,2e localized bonds are no different from those in the valence shell of the neon atom. One can rotate these four bonds into a set of symmetry-preserving (s, p_*x*_, p_*y*_, p_*z*_) -like functions, the p manifold aligning with the three orthogonal *C*_2*v*_ axes of the tetrahedron. These orbitals (although considerably mixed) can also be easily discerned in the canonical MO set. This description is similar to that found when considering superatoms,^37^ and shows how this last concept is even preserved after electron correlation is taken into account.

A final example is the quintet state of Li_4_, an example of the so-called ferromagnetic bonding,^38^ that nicely corroborates that Lewis pairs are not needed for chemical bonds. Although the quintet state is not the ground state of Li_4_, it lacks Jahn–Teller distortions and displays a perfect *T*_d_ geometry. Figure 18 discloses how well Linnett’s insights apply even to excited states in polyatomics. Four same-spin electrons form a perfect tetrahedron that interpenetrates the Li_4_ scaffold, leaving a slightly distorted cube. The four electrons bind four Li atoms. Any localization procedure provides four equivalent 3c,1e bonds.

**Figure 18:**
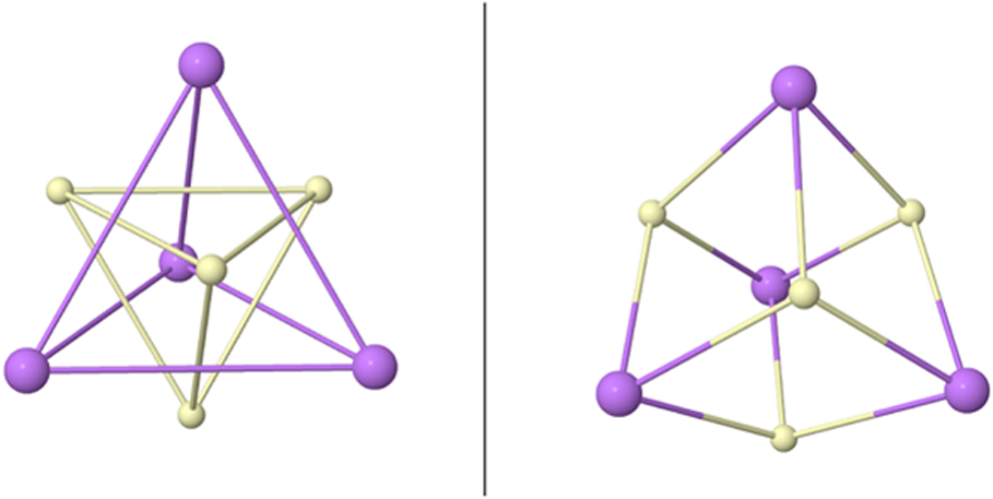
VMC BMs in the *T*_*d*_ quintet state of Li_4_. The overall nuclear-electronic structure is a slightly distorted cube, as seen on the right panel.

## 8 Stereoactivity of Lone Pairs

To end our discussion, we have selected a problem where the BM perspective sheds light on an electronic structure dilemma but where the generalized LDQ model shows its limits. Some AX_6_E molecules (where E denotes a classical lone pair), like BrF_6_^–^ and SeCl_6_^2–^ are known to be exceptions to the general valence shell electron pair repulsion (VSEPR) rules,^39^ showing octahedral structures while they should show distorted geometries like those found in SF_6_^2–^. These are typically called stereoelectronically inactive electron pairs, and have been shown to play a very relevant role in establishing the crystalline structures of many compounds.^40^

Figure 19 displays the BMs of SeF_6_^2–^ and SeCl_6_^2–^. As in our previous SF_6_ example, Figure 15 middle panel, we stress the ionic, nonhypervalent nature of the Se-X interactions. The nature of the inactive lone pair in SeCl_6_^2–^ is very clearly revealed: the two electrons of the pair lie on opposite sides of the Se atom rather than occupying the same region of the space, as in SeF_6_^2–^. There are several frustrated possibilities in which they align along the *C*_3*v*_ axes, but their average is of A_1g_ symmetry, behaving thus similarly to an s-like state. This agrees with one of the justifications for their stereoinactivity: they are deep-lying s states that do not hybridize. Notice, however, that the intuitive geometric explanation provided by Gillespie^39,41^ is also appealing. In SeF_6_^2–^ the electrons of the pair engage in too large Pauli repulsions with the fluoride electrons thus opening room to avoid this intramolecular “steric clash” decreasing the symmetry of the system. Expansion of the Se-X distance on going to the chloride moiety solves this problem and the system recovers *O*_*h*_ symmetry.

**Figure 19:**
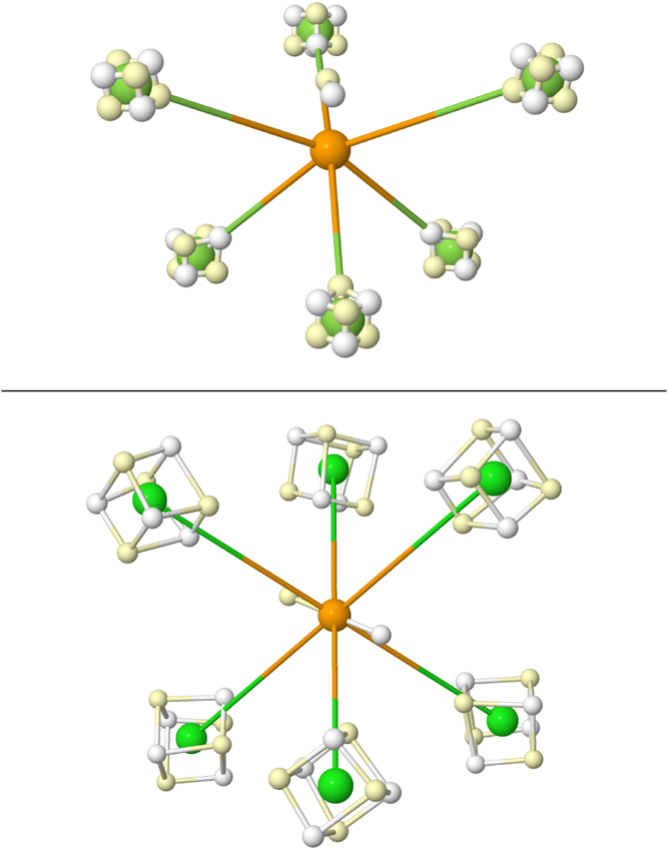
VMC BMs in SeF_6_^2–^ (top) and SeCl_6_^2–^ (bottom). The first shows a classical lone pair, while the second a stereochemically inactive one.

## 9 Conclusions

Contemporary chemists base their daily work on a one-electron picture that owes much to the insights of Robert S. Mulliken and his molecular orbital method. However, in a blatant contradiction, they still cling to the venerable Lewis-Langmuir rules and treat the electron pair as a sacred concept, ignoring many of the fundamental results of quantum mechanics, among which we emphasize the dominant role of antisymmetry in the construction of the electronic structure of molecules. Beginning in 1960, John Wilfred Linnett proposed a modification of the Lewis-Langmuir rules that included, for the first time, an explicit consideration of spin. According to Linnett, the octet should be replaced by a double quartet of electrons with the same spin. The LDQ model was able to beautifully explain the structure of molecules that struggled in the Lewis-Langmuir paradigm, but fell into oblivion with Linnett’s early death and the rise of predictive computational chemistry. Today, after electrons have been naively replaced by ”orbitals”, few textbooks mention the power of the LDQ model.

We have shown here that state-of-the-art computations revindicate the LDQ after some corrections, based on knowledge unavailable in Linnett’s time, are incorporated into the model. Asking about the position of electrons in a molecule is a perfectly valid question in quantum mechanics once it is reformulated in a probabilistic language. The most likely position of the *N* electrons of a system can be obtained by maximizing the square of the wave function in what we call the Born maximum. A study of BMs in atoms and molecules shows that same-spin electron blocks are extremely rigid and that the electronic structure of atoms can be easily interpreted using a generalized LDQ model. Since the electron distribution of ground (or low-lying excited) states of atoms persists in molecules, the LDQ rules have to be slightly modified.

Equipped with the generalized LDQ rules, we have shown how all kinds of chemical bonds, covalent, polar-covalent, ionic, dative, and electron-deficient, can be beautifully yet rigorously described from a perspective very close to a chemist’s expectations. Moreover, the effects of electron correlation, typically ignored by chemists, are directly incorporated into this description. In this way, limitations in our way of thinking are revealed. For example, we have convincingly shown that the electrons involved in multiple 3c,2e bonds in some boron hydrides are highly correlated with each other, so that a description in terms of independent bonds is flawed. Overall, the generalized LDQ ideas presented here offer a fresh view of chemical bonding that we believe can be used in both teaching, laboratory, and theoretical environments.

## Supplementary Material



## Data Availability

The data supporting this article have been included as part of the Supporting Information.

## Notes

Expansion of a multideterminant wave function in terms of two orbital bases related by an unitary transformation (PDF)
