# Supplementary material for: Linnett is Back: Chemical Bonding through the Lens of Born Maxima
Source: J Chem Theory Comput. 2025 Feb 21;21(5):2448–61. doi: 10.1021/acs.jctc.4c01785 (PMC12224293; doi:10.1021/acs.jctc.4c01785)
Supplement: Supplementary file 1 [file ct4c01785_si_001.pdf]

Supporting Information

Linnett is back: chemical bonding through the lens of Born maxima

María Menéndez Herrero, Evelio Francisco, and Ángel Martín Pendás*

Departamento de Química Física y Analítica. Facultad de Química. Universidad de Oviedo. 33006 Oviedo. Spain.

E-mail: ampendas@uniovi.es

Contents

S1 Methodological details **S2**

S1.1 The Slater-Jastrow wavefunction S2

S1.2 Optimization of the wavefunction with *Amolqc* S3

S1.3 Search for maxima of $|\Psi|^2$ S4

S2 Analytical derivatives of $|\Psi|^2$ **S5**

S3 Born maxima in selected ground and low-lying excited states of atoms **S9**

S4 Born maxima in selected ground state diatomics **S9**

S5 Electron coordinates at the HF+J Born maximum in atoms **S10**

S6 Electron coordinates at the HF+J Born maximum in molecules **S21**

S1 Methodological details

S1.1 The Slater-Jastrow wavefunction

An efficient and compact form of including electron correlation effects in Quantum Monte Carlo (QM) calculations is by multiplying a Slater determinant or a linear combination of Slater determinants Φ by a Jastrow factor e^U according to Eq. S1

$$\Psi = \Phi e^U. \tag{S1}$$

$\Phi = \Phi(\mathbf{r}_1, \ldots, \mathbf{r}_N)$ and U are respectively antisymmetric and symmetric functions of the cartesian coordinates of the N electrons of the system, $\mathbf{r}_1, \ldots, \mathbf{r}_N$. U is usually expressed as a linear combination of many-body terms. Many different forms of the Jastrow factor have been used in the literature. Here we use either the Schmidt and Moskowitz (sm) ansatz,[1] or the generalized double exponential (de) one defined by Lüchow and coworkers.[2] In the first case,

$$U = \sum_{A,i<j} U_{Aij}, \tag{S2}$$

where the symbol A stands for nuclei, and i and j represent electrons. Each term U_{Aij} of Eq. S2 has the expression

$$U_{Aij} = \sum_{k}^{N(A)} \Delta(m_{kA}, n_{kA}) c_{kA} \left(\bar{r}_{iA}^{m_{kA}} \bar{r}_{jA}^{n_{kA}} + \bar{r}_{jA}^{m_{kA}} \bar{r}_{iA}^{n_{kA}} \right) \bar{r}_{ij}^{o_{kA}}. \tag{S3}$$

The k sum runs over the $N(A)$ terms used to express the correlation function of atom A, where the c_{kA} are variational parameters that are optimized and m_{kA}, n_{kA} and o_{kA} are predefined integers. The $\bar{r}$ functions represent scaled nuclear-electron and electron-electron distances, described by Eqs. S4 and S5, respectively

$$\bar{r}_{iA} = \frac{a_A r_{iA}}{1 + a_A r_{iA}}, \tag{S4}$$

$$\bar{r}_{ij} = \frac{b_A r_{ij}}{1 + b_A r_{ij}}, \tag{S5}$$

where r_{iA} is the distance between electron i and nucleus A, and r_{ij} the distance between electrons i and j. The parameters m_{kA}, n_{kA} and o_{kA} are integers, and the function Δ is one for $m \neq n$ and $\frac{1}{2}$ for $m = n$. Finally, a_A and b_A are optimizable parameters which in this work are taken as $a_A = b_A = 1$.

In the double exponential ansatz, $U = U_{ee} + U_{en} + U_{een}$. U_{ee} and U_{en} are written as power series of scaled nuclear-electron and electron-electron distances ($U = \sum_k c_k \bar{r}^k$) with

fixed linear terms because the orbitals employed satisfy at least approximately the cusp conditions, and optimizable parameters starting at $k = 2$. The U_{een} term is written as a power series of scaled $ee(r_{ij})$, and $en(r_i)$ coordinates symmetric with respect to particle exchange. In this work we have used a de444 ansatz terminating the series at the quartic power $k = 4$ for both the ee, en, and een terms. This means that in de444 calculations we need to specify 3 parameters for the U_{ee} term, 3 parameters for each type of nucleus to build the U_{en} contribution, and 7 U_{een} parameters per nuclear type, since the number homogeneous symmetric three-body polynomials of degree 4 is 7, as found in Table S1.

We report here the optimized parameters of all the systems studied. The notation is direct in the case of sm ansatzes. In order to save space, we directly write the coefficients in the de444 cases: A first line that contains the 3 U_{ee} coefficients is followed by as many lines as different nuclear types with their corresponding 3 U_{en} coefficients. Finally, we write seven lines with as many columns as the number of nuclear types with the U_{een} coefficients ordered as in Table S1.

Table S1: Three-body polynomials of degree p=4

p	n	
4	7	$r_i^3 r_j + r_i r_j^3$
		$r_i^2 r_j^2$
		$r_{ij}(r_i^3 + r_j^3)$
		$r_{ij}(r_i^2 r_j + r_i r_j^2)$
		$r_{ij}^2(r_i^2 + r_j^2)$
		$r_{ij}^2 r_i r_j$
		$r_{ij}^3(r_i + r_j)$

S1.2 Optimization of the wavefunction with *Amolqc*

In this work, a Slater-Jastrow wavefunction ansatz has been used according to Eq. S1 in variational Monte Carlo (VQMC) calculations. The prefactor Φ was obtained at the HF/cc-pVDZ level of theory using the GAMESS program[3]. This is a single Slater determinant in the case of ^1S atomic states or closed systems, and a symmetry-adapted intra-shell complete active space (CAS) linear combination of Slater determinants in the

case of open-shell atoms. Thus, the non-Jastrow VQMC exact energy should replicate that of the standard electronic structure code if no other correction (e.g. a cusp correction) is applied.

The optimization of the Jastrow factors was performed through the *Amolqc* QMC code[4]. In a first step, samples of 1500 walkers were used, after which an optimization with respect to the variance of the energy was carried out. A new sample of 1500 walkers is then generated using as trial wavefunction the one containing the new parameters, which is followed by another optimization, this time with respect to the energy. The reference energies used in each case were those obtained from the HF/cc-pVDZ calculation for each system.

S1.3 Search for maxima of $|\Psi|^2$

Instead of proceeding with the search of the maxima of $|\Psi|^2$, it is more efficient (and equivalent) to perform the minimization of the function $-\ln(|\Psi|^2)$. Both this minimization and the sampling of $|\Psi|^2$ to generate the $\{\mathbf{r}_i\}$ $(i = 1, \ldots, N)$ electronic distributions are also performed with the *Amolqc* program[4]. The variational Monte Carlo (VMC) method is used to sample $|\Psi|^2$ in the regions where it is large via the Metropolis-Hastings algorithm[5;6]. Then, the algorithms to perform a local minimization of $-\ln(|\Psi|^2)$ are invoked. To reduce the computational cost of these local minimizations, a combination of two methods is used. First, the steepest descent (SD) algorithm is applied and, after five steps, the code is switched to the L-BFGS algorithm[7] to avoid the well known size-step problem of SD.

S2 Analytical derivatives of $|\Psi|^2$

Let $\Psi(1,N)$ a real $N-$electron multideterminant wave function made of n $\alpha-$type electrons and $(N-n)$ $\beta-$type electrons

$$\Psi(1,N)=\sum_k c_k D_k(1,N). \tag{S6}$$

We describe in this subsection of the SI the method used in PROMOLDEN,[8] to find the critical points (CP) of Ψ^2 or, in other words, the positions of the $\alpha-$type electrons $\boldsymbol{r}_\alpha \equiv (\boldsymbol{r}_1,\ldots,\boldsymbol{r}_n)$ and $\beta-$type electrons $\boldsymbol{r}_\beta \equiv (\boldsymbol{r}_{n+1},\ldots,\boldsymbol{r}_N)$ that make $\boldsymbol{\nabla}\left[\Psi^2\right]=\boldsymbol{0}$. We are particularly interested in the maxima of Ψ^2 (Born maxima) and their Hessians. Given that most optimization algorithms deal with the minimization of a function, we pursue to minimize $F=-\frac{1}{2}\ln\left[\Psi^2\right]$. Since $\boldsymbol{\nabla}F=-\boldsymbol{\nabla}\Psi/\Psi$, the CPs are defined by $\boldsymbol{\nabla}\Psi=\sum_k c_k\boldsymbol{\nabla}D_k=0$. After finding a CP of Ψ, we can check whether a minimum of F has been found or not calculating the Hessian

$$\boldsymbol{H}=\boldsymbol{\nabla}\otimes\boldsymbol{\nabla}F=-\frac{(\boldsymbol{\nabla}\otimes\boldsymbol{\nabla}\Psi)}{\Psi}+\frac{(\boldsymbol{\nabla}\Psi)\otimes(\boldsymbol{\nabla}\Psi)}{\Psi^2}. \tag{S7}$$

Following the spin-free formalism, commonly employed in Quantum Monte Carlo (QMC) simulations, each determinant D_k in Eq. S6 is factorized as the product of two derminants D_k^α and D_k^β depending, respectively, on $\boldsymbol{r}_\alpha$ and $\boldsymbol{r}_\beta$

$$\Psi(1,N)=\sum_k^{N_{\rm det}} c_k D_k^\alpha(\boldsymbol{r}_\alpha)D_k^\beta(\boldsymbol{r}_\beta). \tag{S8}$$

When the above summation over k is carried out, the same D_k^α determinant is repeated with many different D_k^β's, or the other way around. This allows Ψ to be written in the form

$$\Psi=\boldsymbol{D}_\alpha^t\boldsymbol{C}\boldsymbol{D}_\beta=\sum_i^{N_{\rm det}^\alpha}\sum_j^{N_{\rm det}^\beta} D_{i,\alpha}C_{ij}D_{j,\beta} \tag{S9}$$

where $\boldsymbol{D}_\alpha$ and $\boldsymbol{D}_\beta$ are column vectors of lengths $N_{\rm det}^\alpha$ and $N_{\rm det}^\beta$, respectively, that contain only the determinants of both spins that are strictly different, and $\boldsymbol{C}$ is the (generally sparse) $N_{\rm det}^\alpha\times N_{\rm det}^\beta$ matrix of c_k coefficients. From Eq. S9,

$$\Psi_{a_p}=\sum_i^{N_{\rm det}^\alpha}\sum_j^{N_{\rm det}^\beta}\left[\left(D_{i,\alpha}\right)_{a_p}C_{ij}D_{j,\beta}+D_{i,\alpha}C_{ij}\left(D_{j,\beta}\right)_{a_p}\right], \tag{S10}$$

where $\Psi_{a_p}\equiv\partial\Psi/\partial a_p$, $a=x,y,z$, $p=1,\ldots,N$, and $(D)_{a_p}$ stands for the first derivative of D with respect to a_p. Since $\boldsymbol{D}_\alpha$ and $\boldsymbol{D}_\beta$ depend only on the coordinates of electrons

1 to n and $n+1$ to N, respectively, only the first (second) term in the above equation is non-zero when Ψ is differentiated with respect to a_p if $1 \leq p \leq n$ $(n+1 \leq p \leq N)$. This means that $\boldsymbol{\nabla}\Psi$ can be written as $\boldsymbol{\nabla}\Psi \equiv \boldsymbol{g} = (\boldsymbol{g}_\alpha, \boldsymbol{g}_\beta)$, where $\boldsymbol{g}_\alpha$ and $\boldsymbol{g}_\beta$ are vectors of $3n$ and $3(N-n)$ elements, respectively, defined by

$$(\boldsymbol{g}_\alpha) \;=\; \left(\boldsymbol{D}_\alpha^t\right)_{a_p} \boldsymbol{C}\boldsymbol{D}_\beta, \quad p=1,\ldots,n \tag{S11}$$

$$(\boldsymbol{g}_\beta) \;=\; \boldsymbol{D}_\alpha^t \boldsymbol{C} \left(\boldsymbol{D}_\beta\right)_{a_p} \quad p=n+1,\ldots,N. \tag{S12}$$

Calling D one of the determinants in $\boldsymbol{D}_\alpha$, formed by the orbitals $u_1,\ldots,u_n$, one has

$$D_{a_p} = \begin{bmatrix} u_1(\boldsymbol{r}_1) & \ldots & u_1(\boldsymbol{r}_p)_{a_p} & \ldots & u_1(\boldsymbol{r}_n) \\ \vdots & & \vdots & & \vdots \\ u_n(\boldsymbol{r}_1) & \ldots & u_n(\boldsymbol{r}_p)_{a_p} & \ldots & u_n(\boldsymbol{r}_n) \end{bmatrix}, \tag{S13}$$

where $u_i(\boldsymbol{r}_p)_{a_p} \equiv (\partial u_i(\boldsymbol{r}_p)/\partial a_p)$, that differs from D only in column p. Assuming that D is non-singular, and that $\det(D)$ and D^{-1} have been calculated beforehand, $\det(\partial D/\partial a_p)$ can be obtained using the Sherman-Morrison (SM) formula

$$\det(\partial D/\partial a_p) = \lambda \det(D), \quad \text{with} \tag{S14}$$

$$\lambda = \left[1 + \text{dot\_product}(D^{-1}(p,:), d_p)\right] \quad \text{and} \tag{S15}$$

$$d_p = [u_1(\boldsymbol{r}_p)_{a_p} - u_1(\boldsymbol{r}_p), \ldots, u_n(\boldsymbol{r}_p)_{a_p} - u_n(\boldsymbol{r}_p)]^t, \tag{S16}$$

with analogous expressions for the β set of electrons. Once $\boldsymbol{\nabla}\Psi$ is known, the second term of $\boldsymbol{H}$ in Eq. S7 is simply the outer product of $\boldsymbol{\nabla}\Psi$ with itself divided by Ψ^2. This term is zero at a CP. To obtain $\boldsymbol{\nabla} \otimes \boldsymbol{\nabla}\Psi$, each component of $\boldsymbol{g}$ must be derived with respect to b_q $(b = x, y, z;\; q = 1, \ldots, N)$. Considering again that $\boldsymbol{D}_\alpha$ and $\boldsymbol{D}_\beta$ depend only on the coordinates of electrons 1 to n and $n+1$ to N, respectively, $\boldsymbol{\nabla} \otimes \boldsymbol{\nabla}\Psi$ has the structure

$$\boldsymbol{\nabla} \otimes \boldsymbol{\nabla}\Psi = \begin{pmatrix} \boldsymbol{h}_{\alpha\alpha} & \boldsymbol{h}_{\alpha\beta} \\ \boldsymbol{h}_{\beta\alpha} & \boldsymbol{h}_{\beta\beta} \end{pmatrix}, \tag{S17}$$

where $\boldsymbol{h}_{\sigma\sigma}$ $(\sigma = \alpha, \beta)$ involve derivatives only with respect to (wrt) coordinates of σ electrons, whereas $\boldsymbol{h}_{\alpha\beta}$ and $\boldsymbol{h}_{\beta\alpha}$ mix derivatives wrt coordinates of electrons with both spins. Explicitly,

$$\boldsymbol{h}_{\alpha\alpha} \;=\; \partial \boldsymbol{g}_\alpha/\partial b_q = \left(\boldsymbol{D}_\alpha^t\right)_{a_p b_q} \boldsymbol{C}\boldsymbol{D}_\beta, \tag{S18}$$

$$\boldsymbol{h}_{\alpha\beta} \;=\; \partial \boldsymbol{g}_\alpha/\partial b_q = \left(\boldsymbol{D}_\alpha^t\right)_{a_p} \boldsymbol{C} \left(\boldsymbol{D}_\beta\right)_{b_q}, \tag{S19}$$

$$\boldsymbol{h}_{\beta\alpha} \;=\; \partial \boldsymbol{g}_\beta/\partial b_q = \left(\boldsymbol{D}_\alpha^t\right)_{b_q} \boldsymbol{C} \left(\boldsymbol{D}_\beta\right)_{a_p}, \tag{S20}$$

$$\boldsymbol{h}_{\beta\beta} \;=\; \partial \boldsymbol{g}_\beta/\partial b_q = \boldsymbol{D}_\alpha^t \boldsymbol{C} \left(\boldsymbol{D}_\beta\right)_{a_p b_q}, \tag{S21}$$

where $\boldsymbol{A}_v$ and $\boldsymbol{A}_{vw}$ (with $\boldsymbol{A} = \boldsymbol{D}^t_\alpha$ or $\boldsymbol{A} = \boldsymbol{D}_\beta$) stand for the first derivative of $\boldsymbol{A}$ with respect to v, and the second derivative of $\boldsymbol{A}$ with respect to v and w, respectively. The intervals of p and q in Eqs. S18−S21 are $p, q = 1, \dots, n$ ($\boldsymbol{h}_{\alpha\alpha}$), $p = 1, \dots, n; q = n{+}1, \dots, N$ ($\boldsymbol{h}_{\alpha\beta}$), $p = n + 1, \dots, N; q = 1, \dots, n$ ($\boldsymbol{h}_{\beta\alpha}$), and $p, q = n + 1, \dots, N$ ($\boldsymbol{h}_{\beta\beta}$). In the computation of $\boldsymbol{h}_{\alpha\alpha}$, $\boldsymbol{h}_{\alpha\beta}$, $\boldsymbol{h}_{\beta\alpha}$, and $\boldsymbol{h}_{\beta\beta}$ we can distinguish several cases depending on the values of a, b, p and q.

1. ($p = q$). Since a single electron is involved, these terms appear in $\boldsymbol{h}_{\alpha\alpha}$ and $\boldsymbol{h}_{\beta\beta}$. If $p \le n$ (block $\boldsymbol{h}_{\alpha\alpha}$), we have $(\boldsymbol{h}_{\alpha\alpha})_{a_pb_p} = (\boldsymbol{D}^t_\alpha)_{a_pb_p} \boldsymbol{C} \boldsymbol{D}_\beta$, where $(\boldsymbol{D}^t_\alpha)_{a_pb_p}$ is the row vector of length N^α_{det} built with the second derivatives with respect to a_p and b_p of the N^α_{det} α determinants. Each of its elements takes the form given by Eq. S13, with $\partial u_i(\boldsymbol{r}_p)/\partial a_p$ replaced by $\partial^2 u_i(\boldsymbol{r}_p)/\partial a_p \partial b_q$ $(i = 1, n)$, i.e.

$$D_{a_pb_p} = \begin{bmatrix} u_1(\boldsymbol{r}_1) & \dots & \partial^2 u_1(\boldsymbol{r}_p)/\partial a_p \partial b_p & \dots & u_1(\boldsymbol{r}_n) \\ \vdots & & \vdots & & \vdots \\ u_n(\boldsymbol{r}_1) & \dots & \partial^2 u_n(\boldsymbol{r}_p)/\partial a_p \partial b_p & \dots & u_n(\boldsymbol{r}_n) \end{bmatrix}. \tag{S22}$$

   Provided that $\det(D) \neq 0$, $\det(D_{a_pb_p}) = \lambda \det(D)$, where λ is given by Eq. S15, with d_p given now by $d_p = [(\partial^2 u_1(\boldsymbol{r}_p)/\partial a_p \partial b_p) - u_1(\boldsymbol{r}_p), \dots, (\partial^2 u_n(\boldsymbol{r}_p)/\partial a_p \partial b_p) - u_p(\boldsymbol{r}_p)]^t$. When $p > n$ (block $\boldsymbol{h}_{\beta\beta}$), we have $(\boldsymbol{h}_{\beta\beta})_{a_pb_p} = \boldsymbol{D}^t_\alpha \boldsymbol{C} (\boldsymbol{D}_\beta)_{a_pb_p}$, with an expression for each of the N^β_{det} matrices in $(\boldsymbol{D}_\beta)_{a_pb_p}$ analogous to Eq. S22, whose determinant can be obtained as in the α case.

2. [($p \le n, q > n$) or ($p > n, q \le n$)]. These terms appear in $\boldsymbol{h}_{\alpha\beta}$ and $\boldsymbol{h}_{\beta\alpha}$. This is the simplest case, since all the required determinant have already been computed.

3. ($p \neq q$) and [($p \le n, q \le n$) or ($p > n, q > n$)]. The elements with ($p \le n$, $q \le n$) and ($p > n$, $q > n$) appear in $\boldsymbol{h}_{\alpha\alpha}$ and $\boldsymbol{h}_{\beta\beta}$, respectively, and are the most expensive to evaluate since they involve derivatives with respect to coordinates of two electrons. Considering the case ($p \le n$, $q \le n$) (the case ($p > n$, $q > n$) is treated analogously), only the determinants in $\boldsymbol{D}_\alpha$ have non-zero derivatives. For instance,

$$D_{a_pb_q} = \begin{bmatrix} u_1(\boldsymbol{r}_1) & \dots & u_1(\boldsymbol{r}_p)_{a_p} & \dots & u_1(\boldsymbol{r}_q)_{b_q} & \dots & u_1(\boldsymbol{r}_n) \\ \vdots & \ddots & \vdots & \ddots & \vdots & \ddots & \vdots \\ u_n(\boldsymbol{r}_1) & \dots & u_n(\boldsymbol{r}_p)_{a_p} & \dots & u_n(\boldsymbol{r}_q)_{b_q} & \dots & u_n(\boldsymbol{r}_n) \end{bmatrix}, \tag{S23}$$

   and $\det(D_{a_pb_q})$ can be computed in two different ways depending on whether or not $\det(\partial D/\partial a_p)$ (Eq. S14) is zero. In the first case, the `lapack` routine `dgetrf` is used

in PROMOLDEN. In the second, the inverse of $(\partial D/\partial a_p)$ (say $(D_{a_p})^{-1}$) is firstly obtained from D^{-1} by using the modified SM formula $(D_{a_p})^{-1} = D^{-1} - \lambda^{-1}\,[D^{-1}d_p D^{-1}(p,:)]$, with λ and d_p given by Eqs. S15 and S16, and then the SM formula is again used

$$\det(D_{a_p b_p}) = \lambda_1\, D_{a_p}, \quad \text{with} \tag{S24}$$

$$\lambda_1 = \left[1 + \text{dot\_product}((D_{a_p})^{-1}(q,:), d_q)\right], \tag{S25}$$

$$d_q = [u_1(\boldsymbol{r}_q)_{b_q} - u_1(\boldsymbol{r}_q), \ldots, u_n(\boldsymbol{r}_q)_{b_q} - u_n(\boldsymbol{r}_q)]^t. \tag{S26}$$

With $\boldsymbol{\nabla} F$ and $\boldsymbol{H}$ available, the function F is minimized in PROMOLDEN using a Limited-memory Broyden-Fletcher-Goldfarb-Shanno (LBFGS) algorithm. In the minimization process, the electron coordinates $(x_1, y_1, z_1, \ldots, x_N, y_N, z_N) \equiv \boldsymbol{x}$, are propagated according to $\boldsymbol{x}_{k+1} = \boldsymbol{x}_k - \alpha_k \boldsymbol{H}_k^{-1} \boldsymbol{\nabla} F(\boldsymbol{x}_k)$, where α_k is a step size, customary determined through a line search method, $\boldsymbol{H}_k^{-1}$ represents the approximate inverse Hessian matrix at the $k-$th iteration, and $\boldsymbol{x}_0$ is vector with the initial guess electron coordinates. LBFGS does not compute $\boldsymbol{H}$ or $\boldsymbol{H}_k^{-1}$ directly because doing so is computationally expensive for large $\boldsymbol{x}$ vectors. Instead, it approximates $\boldsymbol{H}_k^{-1}$ using limited-memory updates derived from the gradient and $\boldsymbol{x}$ vectors over the last m iterations, where m is a small integer $(5-20)$. Once the electronic coordinates remain almost constant from one cycle to the next, PROMOLDEN obtains the final $\boldsymbol{H}$ with the previously derived expressions. Curvatures with different flavours are also computed PROMOLDEN by diagonaling different sub-blocks of the final $\boldsymbol{H}$ matrix. Finally, it is also possible to fix a subset of the electronic coordinates contained in $\boldsymbol{x}$ and maximize Ψ^2 only with respect to the others.

S3 Born maxima in selected ground and low-lying excited states of atoms

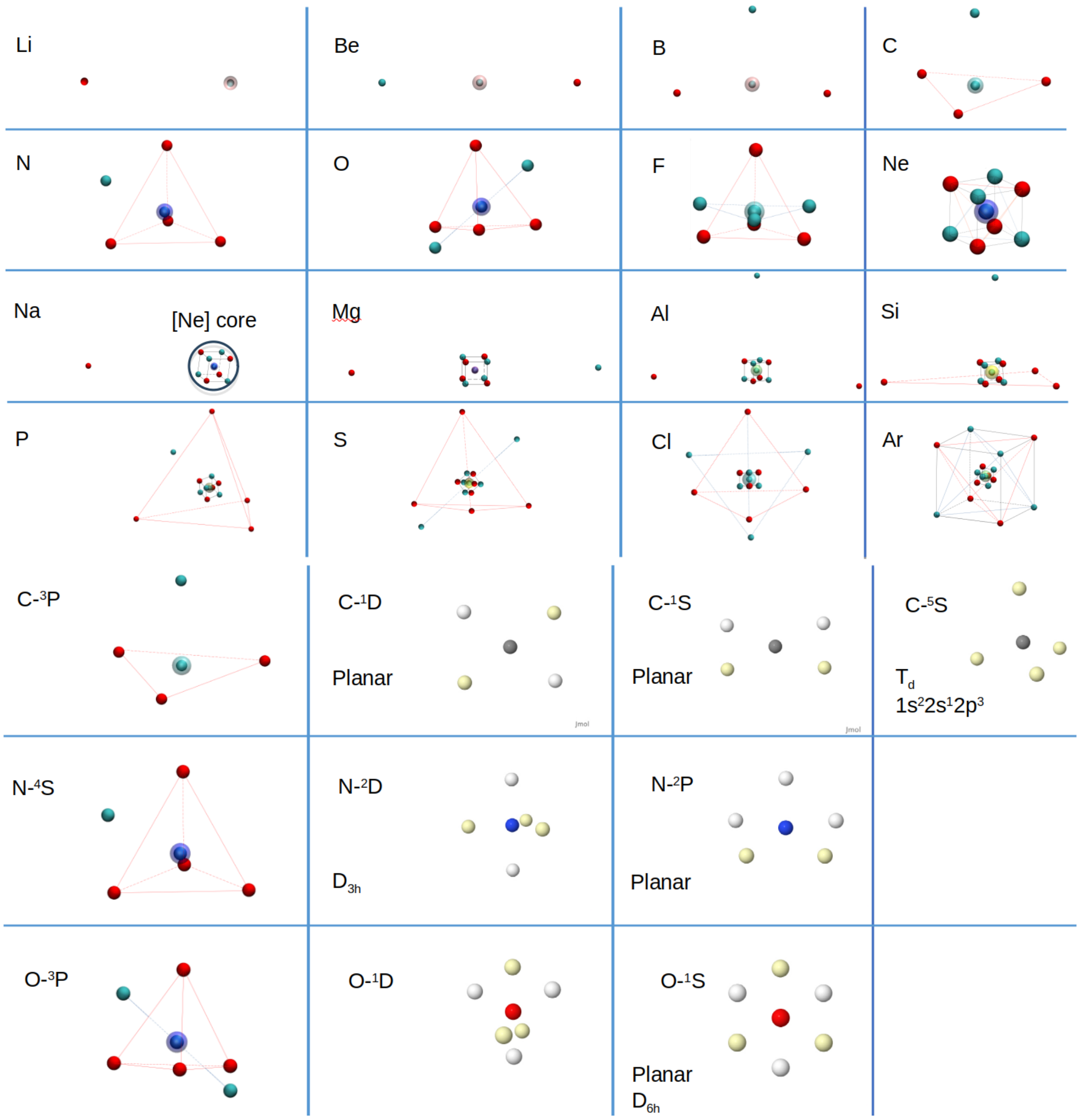

S4 Born maxima in selected ground state diatomics

CASSCF+Jastrow

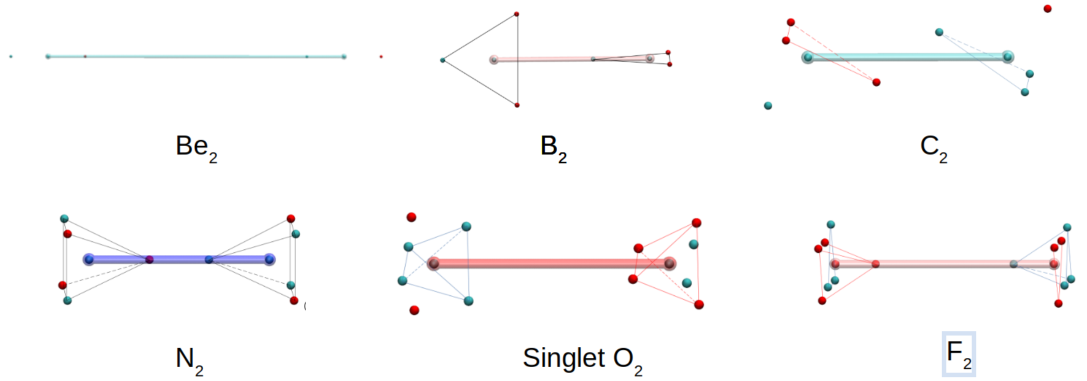

S5 Electron coordinates at the HF+J Born maximum in atoms

Nuclei at the origin of the reference System. Majority spin electrons followed by minority spin ones. All data in au.

Li

0.000000	0.000000	0.000000
0.421021	0.465077	-1.604987
0.000000	0.000000	0.000000

Be

0.000000	0.000000	0.000000
-1.040667	0.317501	-0.275763
1.059810	-0.263955	-0.258784
0.000000	0.000000	0.000000

B

0.000007	-0.000006	-0.817714
0.000001	-0.000004	0.817680
0.000000	0.000000	0.000000
0.841612	0.000154	0.000075
0.000000	0.000000	0.000000

C

0.550702	0.000897	-0.285043
0.000000	0.000000	0.000000
-0.028222	-0.000045	0.619002
-0.522495	-0.000852	-0.333868
0.000000	0.000000	0.000000
-0.547411	-0.394533	-0.000032

N

0.452959	0.102266	-0.162067
-0.284045	-0.131542	-0.379356
0.021393	-0.368988	0.324478
0.000000	0.000000	0.000000
-0.190307	0.398264	0.216945
0.000000	0.000000	0.000000
-0.512166	-0.031832	0.228844

O

0.000000	0.000000	0.000000
0.122054	0.219704	-0.315277
0.124408	0.174560	0.341495
-0.407731	-0.024291	-0.000181
0.156534	-0.370646	-0.026044
0.442312	0.001004	-0.000001
0.000000	0.000000	0.000000
-0.442312	-0.001004	-0.000000

F

0.000000	0.000000	0.000000
-0.011966	0.331751	0.103234
-0.278540	-0.169014	0.120292
0.288161	-0.149481	0.123338

0.002313	-0.013118	-0.344136
0.166533	0.320455	-0.000000
-0.360440	-0.015811	0.000000
0.000000	0.000000	0.000000
0.193880	-0.304648	-0.000000

Ne

0.290334	0.089594	-0.022201
-0.049682	-0.115557	0.277475
-0.056950	-0.212889	-0.210355
-0.183702	0.238851	-0.044919
0.000000	0.000000	0.000000
-0.100984	0.021407	-0.286632
-0.100738	-0.259376	0.124056
0.000000	0.000000	0.000000
-0.102928	0.236677	0.161875
0.304650	0.001291	0.000701

Na

0.919778	0.264971	1.834583
0.218399	-0.145186	0.011813
-0.116465	-0.036245	-0.231858
0.061809	0.253578	0.028129
-0.163573	-0.072098	0.192255
0.000000	0.000000	0.000000
0.110869	-0.177160	0.158491
0.000000	0.000000	0.000000
0.121333	-0.002647	-0.232527
-0.253501	-0.058710	-0.032986
0.021298	0.238517	0.107021

Mg

0.121635	-0.103864	0.175085
0.015915	0.235654	0.021566
0.089911	-0.066568	-0.209112
1.564740	0.507609	-0.104216
-0.226986	-0.065070	0.012430
0.000000	0.000000	0.000000
0.602824	-1.346749	-0.734723
-0.090211	0.197396	0.093853
-0.066529	-0.017096	-0.227019
-0.079766	-0.188211	0.120209
0.000000	0.000000	0.000000
0.236687	0.007504	0.012736

Al

-0.000002	0.000005	-1.601623
-0.037819	0.201314	-0.069832
-0.000004	0.000007	1.601611
0.191749	-0.067387	-0.074291
-0.155746	-0.131876	-0.071993
0.000000	0.000000	0.000000
0.001805	-0.002039	0.215458
1.388305	-0.000232	-0.000133
0.069262	-0.063878	0.195162
0.076242	-0.134917	-0.151518
0.070972	0.200881	-0.039727
0.000000	0.000000	0.000000
-0.215992	-0.002085	-0.003916

Si

-1.222127	-0.004374	0.696204
1.215462	0.004339	0.707789

0.000000	0.000000	0.000000
0.000636	-0.198657	-0.000929
0.178018	0.066805	-0.053980
-0.042307	0.064997	0.181801
0.006707	0.000019	-1.406362
-0.136344	0.066142	-0.126898
-0.162505	-0.112912	0.009832
0.063019	0.009046	-0.188320
0.142510	-0.079014	0.113921
-0.042633	0.183166	0.064568
0.981793	0.707710	0.000249
0.000000	0.000000	0.000000

P

-0.900136	-0.834106	-0.051544
0.181433	0.334942	1.167701
0.000000	0.000000	0.000000
0.121442	0.113125	-0.075454
-0.315216	0.984090	-0.663951
-0.115472	-0.059351	-0.127994
1.033921	-0.484928	-0.452207
0.087066	-0.142111	0.073911
-0.093036	0.088336	0.129537
-0.561304	-0.908175	-0.132059
-0.162649	0.039448	-0.075696
-0.026158	-0.080047	0.163245
0.095764	0.154275	0.022450
0.000000	0.000000	0.000000
0.092778	-0.114104	-0.110062

S

-0.032748	1.067088	-0.004065
-0.066398	0.154661	-0.010311

0.512921	-0.342288	-0.862074
-0.988873	-0.381218	-0.003184
0.010095	-0.067224	-0.154272
0.504898	-0.335911	0.869258
0.000000	0.000000	0.000000
0.154244	0.009438	0.067385
-0.097954	-0.096848	0.097197
-0.000490	0.168286	0.000532
0.062172	-0.056732	0.147494
0.097053	-0.055763	-0.127715
-0.005173	1.028913	-0.000001
0.005163	-1.028947	-0.000001
-0.158731	-0.056850	-0.020318
0.000000	0.000000	0.000000

Cl

0.006245	0.139135	0.071846
0.000000	0.000000	0.000000
0.172873	-0.878089	0.300156
-0.013045	-0.113592	0.107169
0.003906	0.017408	-0.939410
0.666018	0.582194	0.328726
0.130895	-0.021648	-0.083397
-0.843846	0.279758	0.316504
-0.124094	-0.003893	-0.095611
0.000488	-0.000009	-0.158292
0.423117	-0.835677	-0.000010
0.136630	0.055899	0.052836
-0.020151	-0.146405	0.052362
0.000000	0.000000	0.000000
-0.935050	0.050323	-0.000010
0.509932	0.785674	-0.000010
-0.116959	0.090515	0.052047

Ar

-0.705269	0.454947	0.149410
0.446588	0.365853	-0.627228
-0.093518	-0.010466	0.112357
0.133467	0.033431	0.050479
0.000000	0.000000	0.000000
-0.216084	-0.792013	-0.229623
0.005343	-0.127991	-0.071198
-0.045292	0.105025	-0.091639
0.474765	-0.028787	0.707442
-0.074817	-0.001691	-0.126012
0.000000	0.000000	0.000000
0.631119	0.246250	0.517450
0.041187	-0.136432	0.034196
0.230960	-0.709575	-0.412146
-0.161136	0.617323	-0.565378
-0.085116	0.053511	0.106636
0.118746	0.084612	-0.014820
-0.700943	-0.153997	0.460074

K

-0.074786	-0.069978	0.090482
0.681845	-0.253500	0.005088
0.092813	0.075091	0.066060
0.003365	0.707855	0.163714
0.063296	-0.087123	-0.083796
1.461582	1.574323	-1.887796
-0.081405	0.081928	-0.072647
-0.289112	-0.094864	-0.660374
-0.402505	-0.366289	0.499821
0.000000	0.000000	0.000000
-0.057843	-0.074021	-0.099065

-0.006539	-0.593097	0.427923
0.029964	0.124362	-0.047702
-0.666447	0.301135	-0.009479
0.000000	0.000000	0.000000
0.190555	-0.191790	-0.679580
0.482420	0.483736	0.261141
0.114488	-0.061502	0.041823
-0.086609	0.011162	0.104945

Ca

-0.609942	0.262774	0.158904
-0.116839	0.054281	0.019370
0.000000	0.000000	0.000000
2.147029	-0.883466	-0.529224
-0.005431	-0.126414	0.029833
0.333569	0.430233	-0.387565
0.347244	-0.111005	0.560020
0.088088	0.058578	0.075553
0.034006	0.013635	-0.124725
-0.084467	-0.576379	-0.327986
-0.587708	-0.142796	0.284699
0.000000	0.000000	0.000000
0.399228	-1.242187	1.991961
-0.098103	-0.082696	0.020893
-0.022907	0.068018	-0.108730
-0.105807	0.348415	-0.577702
0.110808	-0.066695	-0.013112
0.366732	0.369743	0.418974
0.010166	0.081476	0.100785
0.324272	-0.567507	-0.138612

C ^3P

0.000000	0.000000	0.000000

-0.034353	0.703748	-0.147224
-0.592287	-0.381624	-0.147225
0.626640	-0.322123	-0.147225
0.000000	0.000000	0.000000
0.000000	0.000003	0.787302

C ^1D

0.357355	-0.650564	0.131412
-0.357374	0.650554	-0.131400
0.000000	0.000000	0.000000
0.000000	0.000000	0.000000
0.117279	-0.028323	0.744066
-0.117268	0.028322	-0.744083

C ^1S

0.357355	-0.650564	0.131412
-0.357374	0.650554	-0.131400
0.000000	0.000000	0.000000
0.000000	0.000000	0.000000
0.117279	-0.028323	0.744066
-0.117268	0.028322	-0.744083

C ^5S

0.000000	0.000000	0.000000
0.432474	-0.098941	-0.564080
-0.164835	0.696219	0.055840
-0.601078	-0.381701	-0.089552
0.333438	-0.215577	0.597792
0.000000	0.000000	0.000000

N ^4S

0.000000	0.000000	0.000000

0.432474	-0.098941	-0.564080
-0.164835	0.696219	0.055840
-0.601078	-0.381701	-0.089552
0.333438	-0.215577	0.597792
0.000000	0.000000	0.000000

N ^2D

0.000000	0.000000	0.000000
0.000000	-0.243550	0.551743
0.000000	-0.356049	-0.486791
0.000000	0.599598	-0.064951
0.624407	-0.000000	-0.000000
0.000000	0.000000	0.000000
-0.624407	0.000000	0.000000

N ^2P

0.000000	0.000000	0.000000
0.565847	-0.167031	-0.074060
-0.326819	-0.234523	-0.437899
-0.210668	0.353911	0.451216
0.540331	0.093990	0.297397
-0.600866	0.007707	-0.167739
0.000000	0.000000	0.000000

O ^3P

-0.274330	0.088834	-0.409996
0.274329	-0.409997	-0.088835
0.000000	0.000000	0.000000
0.274330	0.409997	0.088833
-0.274328	-0.088834	0.409997
-0.536659	0.000000	0.000000
0.000000	0.000000	0.000000

0.536659	-0.000000	0.000000

O ^1D

0.242379	-0.332306	-0.318721
0.242366	0.332308	0.318729
0.000000	0.000000	0.000000
-0.513414	-0.000013	0.000001
-0.242371	0.332302	-0.318724
-0.242378	-0.332303	0.318722
0.513418	-0.000007	0.000002
0.000000	0.000000	0.000000

O ^1S

-0.437773	-0.263108	0.085846
0.000000	0.000000	0.000000
0.002794	0.516692	0.035517
0.434978	-0.253584	-0.121363
0.437773	0.263108	-0.085846
-0.434978	0.253585	0.121363
0.000000	0.000000	0.000000
-0.002794	-0.516692	-0.035517

$\mathbf{B}_2$

0.857825	-0.262306	-0.246451
0.000000	0.000000	0.000000
-0.867952	-0.261494	-0.209015
0.000000	0.000000	0.000000
0.014309	0.740111	0.643557

$\mathbf{C}_2$

0.191620	0.622305	-0.824700
0.000000	0.000000	1.197141

```
-0.374842     0.000001     1.877355
 0.000000     0.000000    -1.197141
-0.520638    -0.000080    -1.704170
 0.191690    -0.622219    -0.824603
 0.520650    -0.000109     1.704168
-0.191711    -0.622207     0.824581
-0.191596     0.622314     0.824713
 0.000000     0.000000     1.197141
 0.000000     0.000000    -1.197141
 0.374859     0.000022    -1.877347
```

S6 Electron coordinates at the HF+J Born maximum in molecules

Majority spin electrons followed by minority spin ones.

```
-----------------
b4h4.xyz
-----------------
 -1.2873 -1.2873  1.2873
 -1.2873  1.2873 -1.2873
 -0.6424  0.0404 -0.4990
  0.6005  0.6005  0.6005
  0.6005 -0.6005 -0.6005
  0.6302 -0.0403 -0.4953
  0.9843  0.9743  0.9718
  0.0398  0.6327  0.4964
 -0.6005 -0.6005  0.6005
 -0.6005  0.6005 -0.6005
 -0.0408 -0.6399  0.4978
  0.9746 -0.9840 -0.9724
 -0.6301 -0.0403  0.4952
  1.2873 -1.2873 -1.2873
```

```
 0.6424  0.0404  0.4990
-0.6005  0.6005 -0.6005
-0.0398  0.6327 -0.4964
 0.6005 -0.6005 -0.6005
-0.9746 -0.9840  0.9724
 0.0408 -0.6399 -0.4978
-0.6005 -0.6005  0.6005
-0.9843  0.9743 -0.9718
 1.2873  1.2873  1.2873
 0.6005  0.6005  0.6005
-----------------
c3o2.xyz
-----------------
-0.7846 -0.2267  0.4429
 0.0000 -0.2209 -0.4531
 0.0000  0.0000  0.0383
-1.2087  0.0000  0.4837
 0.7846 -0.2267  0.4430
-2.0576  0.2162  0.9623
 1.2087  0.0000  0.4837
-0.0000  0.5088  0.0178
 2.1782 -0.2153  0.5404
-2.5840  0.2150  0.6682
-2.1782 -0.2152  0.5403
-2.4634 -0.2157  1.0742
-2.2958  0.0000  0.8072
 2.2958  0.0000  0.8072
 2.0575  0.2162  0.9622
 2.5840  0.2149  0.6681
 2.4633 -0.2156  1.0742
-0.7846  0.2267  0.4430
 2.1782  0.2153  0.5403
-2.1781  0.2153  0.5404
```

```
 -1.2087  0.0000  0.4837
 -0.0000  0.2210 -0.4531
  0.0000 -0.5088  0.0178
  1.2087  0.0000  0.4837
  2.4633  0.2156  1.0742
  0.0000  0.0000  0.0383
 -2.0576 -0.2163  0.9622
 -2.5840 -0.2149  0.6681
 -2.2958  0.0000  0.8072
 -2.4634  0.2155  1.0743
  2.5840 -0.2150  0.6682
  2.0575 -0.2162  0.9622
  0.7846  0.2267  0.4430
  2.2958  0.0000  0.8072
-----------------
cao.xyz
-----------------
  0.0418 -0.1430 -0.0495
  2.1066  0.0257 -0.0572
  2.0344 -0.3373  0.2560
  2.0269 -0.0638 -0.0221
  0.2665  0.0773  0.2646
  2.0344 -0.1067 -0.4098
  1.8160  0.3222  0.1116
  2.0111  0.0531  0.0184
  2.1066 -0.0152  0.0609
  0.2665  0.2244 -0.1601
  0.4970 -0.1565 -0.0542
  2.4544  0.1502  0.0520
  2.1143 -0.0535 -0.0185
  2.4545 -0.1501 -0.0520
  0.2664 -0.0773 -0.2646
  0.0419  0.1431  0.0496
```

```
  2.0343  0.3372 -0.2560
  2.0343  0.1067  0.4097
  2.0990  0.0637  0.0221
  2.0190 -0.0255  0.0572
  0.2664 -0.2244  0.1601
  2.0190  0.0153 -0.0607
  0.4971  0.1563  0.0541
  1.8160 -0.3224 -0.1117
-----------------
co2.xyz
-----------------
  0.2514 -0.1241 -1.1863
  0.0030  0.2804 -1.1863
 -0.1311 -0.0822  0.8304
  0.0175  0.2862  1.1225
 -0.2219 -0.1363 -1.2477
 -0.0397 -0.0244 -0.3934
 -0.1496 -0.0938  1.3564
  0.2652 -0.1089  1.1225
 -0.0201 -0.2860 -1.1225
  0.1505  0.0924 -1.3564
 -0.2642  0.1114 -1.1225
 -0.0004 -0.2804  1.1863
 -0.2526  0.1218  1.1863
  0.2206  0.1383  1.2477
  0.0395  0.0247  0.3934
  0.1318  0.0810 -0.8304
-----------------
co.xyz
-----------------
  0.0554  0.1171 -0.3461
  0.2737  0.0280  1.1954
 -0.1139 -0.2407  1.2228
```

```
  0.0000  0.0000  1.1253
  0.0000  0.0000  0.0152
 -0.1519  0.2294  1.1954
 -0.0089 -0.0189  0.4379
  0.0000  0.0000  0.0152
 -0.0573 -0.1211 -0.3482
  0.1478 -0.2320  1.0120
  0.0145  0.0307  1.4091
  0.1100  0.2325  0.9572
  0.0000  0.0000  1.1253
 -0.2731 -0.0329  1.0121
-----------------
kcl.xyz
-----------------
  2.3666 -0.0293 -0.0768
  2.3582 -0.1576 -0.4189
  2.7437  0.2343  0.0646
  2.3517 -0.3416  0.2888
  2.0159  0.2652  0.0658
 -0.4076 -0.0029 -0.0819
  2.2992 -0.0241  0.0393
 -0.3246  0.1930  0.5108
  2.4334 -0.0287  0.0396
 -0.2731 -0.0462 -0.0122
 -0.7585 -0.3314 -0.0862
 -0.3262  0.4191 -0.3500
  2.3702  0.0821 -0.0020
 -0.3402  0.0917  0.0240
  0.1513 -0.2771 -0.0736
 -0.4074 -0.0427  0.0700
 -0.2900 -0.4181  0.3494
 -0.4407  0.0456  0.0120
  2.3749  0.3420 -0.2888
```

```
  2.3647 -0.0821  0.0020
  2.3683  0.0293  0.0768
  2.3011  0.0286 -0.0395
  2.7272 -0.2590 -0.0641
  1.9993 -0.2409 -0.0663
 -0.3739 -0.0918 -0.0240
  2.3682  0.1578  0.4190
 -0.7933  0.2850  0.0757
 -0.2916 -0.1925 -0.5099
 -0.3067  0.0030  0.0821
  0.1183  0.3221  0.0838
  2.4353  0.0242 -0.0393
 -0.3069  0.0430 -0.0702
-----------------
kf.xyz
-----------------
  0.0637  0.1438 -0.1340
  1.8527 -0.2538 -0.2717
 -0.2141 -0.1304 -0.1407
  2.1116  0.0128  0.0791
  2.1450  0.0480 -0.0449
  0.0619 -0.1445  0.1350
  2.0188  0.0173 -0.0160
  1.8528  0.2535  0.2721
 -0.2145  0.1311  0.1397
  2.1115 -0.0781 -0.0182
  2.3655  0.2659 -0.2481
  2.3660 -0.2656  0.2477
  1.8525  0.2718 -0.2535
  2.3658  0.2480  0.2655
  2.3657 -0.2477 -0.2659
  2.1748 -0.0174  0.0162
  0.0626  0.1341  0.1447
```

```
 -0.2134  0.1406 -0.1313
  2.0484 -0.0478  0.0447
  1.8531 -0.2721  0.2539
  2.0818 -0.0128 -0.0791
  0.0630 -0.1349 -0.1436
 -0.2152 -0.1398  0.1302
  2.0819  0.0781  0.0181
-----------------
li3+.xyz
-----------------
 -0.8861  1.5348  0.0000
 -0.8861 -1.5348  0.0000
 -0.4968  0.2463  0.0000
  1.7722  0.0000  0.0000
 -0.8861  1.5348  0.0000
 -0.8861 -1.5348  0.0000
  0.4619 -0.3066 -0.0000
  1.7722  0.0000  0.0000
-----------------
li4.xyz
-----------------
  0.8436  0.8484 -0.8503
 -0.8481 -0.8500 -0.8445
 -0.8472  0.8465  0.8489
  0.8514 -0.8451  0.8459
-----------------
linh3-1-1.xyz
-----------------
 -6.0967  2.3031 -1.2262
 -4.1998  1.5291  0.3505
 -5.3080  1.6508  0.1346
 -5.2044  1.6203  0.4354
 -5.2680  1.8477  0.7118
```

```
 -5.5250  0.7045  0.7238
 -6.0967  2.3031 -1.2262
 -4.8438  1.6062  0.3980
 -5.3691  1.7889  0.2230
 -5.2044  1.6203  0.4354
 -5.3379  1.2987  0.5371
 -5.3440  2.2113  1.2453
-----------------
mgo.xyz
-----------------
  0.8069  0.1718 -0.2155
  2.2830  0.0006 -0.0005
  2.4881 -0.1445 -0.0242
  2.4883  0.0509  0.1373
  0.8066 -0.2726 -0.0412
  2.4892  0.0930 -0.1126
  0.4089  0.0002  0.0001
  0.8069  0.1006  0.2566
  1.0148 -0.0002 -0.0001
  0.6079 -0.1001 -0.2555
  2.3856 -0.0507 -0.1369
  0.6079 -0.1711  0.2146
  2.3847 -0.0928  0.1123
  2.5919 -0.0006  0.0005
  0.6082  0.2715  0.0410
  2.3858  0.1441  0.0241
-----------------
nacl.xyz
-----------------
  2.6513  0.0926 -0.1346
  2.6491  0.0710  0.1479
  2.6514 -0.1629 -0.0120
  0.2017  0.0400 -0.0848
```

```
  2.4200 -0.0007 -0.0014
  0.1655  0.0405  0.0669
  0.5831 -0.4003  0.2419
  0.3125  0.0132  0.0219
  0.2016 -0.0936 -0.0041
 -0.0838 -0.2429 -0.4044
 -0.0875  0.2415  0.4022
  0.5830  0.4017 -0.2398
  2.5339  0.1621  0.0119
  2.5362 -0.0707 -0.1473
  0.5811  0.2416  0.4021
 -0.0856 -0.4039  0.2412
  0.2751 -0.0406 -0.0671
  0.2390 -0.0399  0.0847
 -0.0857  0.4026 -0.2433
  2.5340 -0.0921  0.1339
  2.7673  0.0007  0.0014
  0.1281 -0.0131 -0.0218
  0.2391  0.0936  0.0041
  0.5850 -0.2402 -0.3999
-----------------
naf.xyz
-----------------
  2.0447  0.1571 -0.0271
  2.0440 -0.1020 -0.1222
  0.4575 -0.0000 -0.0000
  0.1326  0.1423  0.1799
  0.1326  0.0846 -0.2131
  2.2730 -0.0003 -0.0006
  2.0449 -0.0548  0.1498
  0.1325 -0.2269  0.0333
  2.1577 -0.1586  0.0273
  0.2960 -0.1416 -0.1790
```

```
  0.2961  0.2258 -0.0331
  2.1584  0.1030  0.1233
 -0.0319  0.0000  0.0000
  0.2960 -0.0842  0.2121
  1.9335  0.0003  0.0005
  2.1575  0.0553 -0.1512
-----------------
no3-.xyz
-----------------
 -6.0004  2.7632 -1.9304
 -7.3434  3.3590 -0.1967
 -7.4211  3.5306 -0.6037
 -5.1574  2.6666 -0.0570
 -5.8003  2.9372 -1.9813
 -5.4323  2.8314  0.2801
 -6.2212  2.8113 -2.0750
 -5.9211  2.5062 -1.9409
 -5.3845  2.6433  0.0901
 -7.1611  3.7532 -0.3126
 -7.2507  3.5129 -0.3971
 -5.4815  2.3919  0.1339
 -6.0897  2.8274 -1.5609
 -5.9841  2.8912 -0.5200
 -6.9342  3.3337 -0.5252
 -6.2119  2.9731 -0.7458
 -6.2119  2.9731 -0.7458
 -5.7402  2.6881 -1.9275
 -7.4176  3.3057 -0.4566
 -5.5115  2.5911  0.3210
 -5.2469  2.4325 -0.0035
 -6.4858  3.1218 -0.6354
 -6.1376  2.9130 -1.0624
 -7.2455  3.7221 -0.5706
```

```
 -5.3845  2.6433  0.0901
 -7.2012  3.5641 -0.1359
 -6.0598  3.0112 -2.0247
 -7.2507  3.5129 -0.3971
 -5.2367  2.8701  0.0998
 -6.0004  2.7632 -1.9304
 -6.1883  2.5688 -1.9640
 -5.6510  2.7216 -0.1654
-----------------
secl6.xyz
-----------------
  1.8633 -0.1541  0.0299
  0.2656  3.0174  0.0626
  0.3920  0.4350 -2.4174
 -0.5423  0.2168 -2.4190
  2.5805  0.2081 -0.5447
  0.3876  2.0284  0.0918
  0.1096 -2.8265  0.4721
  0.3963 -2.5780 -0.4276
 -0.5446 -2.5805 -0.2082
 -2.4190  0.5425 -0.2162
 -2.4175 -0.3916 -0.4354
  0.0298 -1.8633  0.1541
  0.0916 -0.3876 -2.0284
 -0.4352  2.4174  0.3918
  0.1541 -0.0298  1.8633
 -0.3516  0.3517 -0.3517
 -3.0174 -0.0627  0.2656
 -0.2083  0.5446  2.5805
 -0.2165  2.4190 -0.5424
  0.0624 -0.2656 -3.0174
 -0.4275 -0.3964  2.5780
  2.8265 -0.4721  0.1097
```

```
 2.5780  0.4277  0.3962
 0.4721 -0.1095  2.8265
-2.0284 -0.0920  0.3875
-0.1093  0.4722 -2.8265
-0.1541  1.8633 -0.0298
-0.0626 -3.0174 -0.2656
 0.4275  2.5780 -0.3964
-0.3917 -2.4175  0.4352
 3.0174  0.2656 -0.0627
 0.2165 -0.5424  2.4190
-2.5779  0.3962  0.4277
 0.3516 -0.3517  0.3517
 0.2083  2.5805  0.5446
 0.5445 -0.2085 -2.5805
-0.2656  0.0625  3.0174
-1.8633  0.0299 -0.1541
-0.4721  2.8265 -0.1095
-0.3876  0.0918  2.0284
-2.5805 -0.5447  0.2080
-0.0919 -2.0284 -0.3876
-0.3966 -0.4273 -2.5780
 2.4175 -0.4353 -0.3916
 2.0284  0.3875 -0.0920
 0.4351  0.3918  2.4174
 2.4190 -0.2163  0.5425
 0.5424 -2.4190  0.2164
-0.0297  0.1541 -1.8633
-2.8265  0.1098 -0.4721
-----------------
sef6.xyz
-----------------
-1.1829  1.6335 -1.0822
-1.3681  0.1912  1.0773
```

-0.8716	-1.4691	-0.8548
-1.3796	-0.2015	1.0636
0.5876	1.0085	1.0609
0.5143	1.2961	0.7845
-1.0211	1.8530	-0.7930
-0.8160	1.7836	-1.1265
-1.1834	-1.6338	-1.0815
-1.1284	-0.0003	0.7314
0.7813	1.3623	1.0793
0.7816	-1.3623	1.0792
1.9138	-0.2045	-0.8275
1.9149	0.2028	-0.8247
2.1772	0.0000	-1.0555
1.7896	0.0017	-1.1527
0.5827	-1.0113	1.0609
0.8696	1.1018	0.7860
0.5182	-1.2975	0.7810
0.8704	-1.0977	0.7896
-0.8715	1.4692	-0.8547
-1.5866	0.0105	0.8050
-0.8163	-1.7830	-1.1272
-1.0201	-1.8533	-0.7930
0.2846	0.0001	-0.7579
-0.7610	1.7424	-0.8471
-0.9311	-1.5247	-1.1447
-1.0681	-1.8918	-1.0642
-1.1362	-1.5922	-0.8037
-0.9301	1.5250	-1.1448
0.8599	1.0960	1.0702
-0.7605	-1.7420	-0.8479
-1.3931	-0.1967	0.7790
-1.3810	0.2073	0.7928
0.5666	-0.9740	0.7345

```
  0.8562 -1.0947  1.0737
  1.7037 -0.0000 -0.8634
  1.9836  0.2004 -1.1028
 -1.1645 -0.0109  1.0571
  2.1128 -0.0017 -0.7852
  0.7891  1.3748  0.8023
  1.9825 -0.1988 -1.1055
  0.7938 -1.3721  0.8023
  0.5160  1.2862  1.0708
  0.5669  0.9740  0.7344
 -0.3231 -0.0000 -0.7386
 -1.5679  0.0003  1.0828
 -1.1366  1.5916 -0.8044
 -1.0682  1.8917 -1.0643
  0.5152 -1.2901  1.0672
-----------------
sf6.xyz
-----------------
  0.1915  1.6431  0.1358
  0.0476 -0.0559 -0.0707
 -0.1915 -1.6391 -0.1362
  1.8113 -0.1505  0.0004
  1.4147 -0.1235  0.0003
 -0.1919  0.1355 -1.6411
 -0.1004 -0.0169 -0.0052
  1.6411  0.1364  0.1912
  0.0187  0.0989 -0.0166
  0.0003 -0.1235 -1.4147
  1.6411  0.1354 -0.1919
  0.0003 -0.1505 -1.8113
 -1.8113 -0.1505 -0.0000
 -0.0001 -1.8134  0.1479
 -1.6411  0.1360 -0.1915
```

0.0341	-0.0260	0.0925
-0.1916	1.6431	0.1357
-1.4147	-0.1235	-0.0000
-1.6411	0.1359	0.1916
0.0001	1.8092	-0.1530
-0.0001	-1.4168	0.1264
0.1917	-1.6391	-0.1359
0.1913	0.1363	-1.6411
-0.1919	-0.1354	1.6411
0.0003	0.1235	1.4147
0.0004	0.1505	1.8113
0.1912	-0.1364	1.6411
0.0001	1.4126	-0.1206
0.0001	-1.8092	-0.1530
0.1919	-0.1355	-1.6411
0.1916	1.6391	-0.1360
0.0001	-1.4126	-0.1207
0.1004	0.0169	0.0052
-0.0003	0.1505	-1.8113
-0.0003	0.1235	-1.4147
-0.1915	1.6391	-0.1361
1.6411	-0.1354	0.1919
1.6411	-0.1364	-0.1912
1.8113	0.1505	-0.0004
-0.0341	0.0260	-0.0925
-1.6411	-0.1359	-0.1916
-1.8113	0.1505	0.0000
-1.4147	0.1235	0.0000
-1.6411	-0.1360	0.1915
0.1919	0.1354	1.6411
-0.0001	1.8134	0.1479
-0.0476	0.0559	0.0707
1.4147	0.1235	-0.0003

0.1915	-1.6431	0.1359
-0.1912	0.1364	1.6411
-0.1917	-1.6431	0.1356
-0.0001	1.4168	0.1264
-0.0003	-0.1235	1.4147
-0.0187	-0.0989	0.0166
-0.0004	-0.1505	1.8113
-0.1913	-0.1363	-1.6411

References

[1] K. E. Schmidt and J. W. Moskowitz, *The Journal of Chemical Physics*, 1990, **93**, 4172–4178.

[2] A. Lüchow, A. Sturm, C. Schulte and K. Haghighi Mood, *J. Chem. Phys.*, 2015, **142**, 084111.

[3] M. W. Schmidt, K. K. Baldridge, J. A. Boatz, S. T. Elbert, M. S. Gordon, J. H. Jensen, S. Koseki, N. Matsunaga, K. A. Nguyen, S. J. Su, T. L. Windus, M. Dupuis and J. A. Montgomery, *J. Comput. Chem.*, 1993, **14**, 1347–1363.

[4] A. Lüchow, S. Manten, C. Diedrich, A. Bande, T. C. Scott, A. Schwarz, R. Berner, R. Petz, A. Sturm, M. Hermsen, K. H. Mood, C. Schulte, L. Reuter, M. A. Heuer and J. Ludovicy.

[5] N. Metropolis, A. W. Rosenbluth, M. N. Rosenbluth, A. H. Teller and E. Teller, *J. Chem. Phys.*, 1953, **21**, 1087–1092.

[6] W. K. Hastings, *Biometrika*, 1970, **57**, 97–109.

[7] D. C. Liu and J. Nocedal, *Math. Program.*, 1989, **45**, 503–528.

[8] A. Martín Pendás and E. Francisco, PROMOLDEN. A QTAIM/IQA code (Available from the authors upon request).
